# A mathematical framework for measuring and tuning tempo in developmental gene regulatory networks

**DOI:** 10.1242/dev.202950

**Published:** 2024-06-24

**Authors:** Charlotte L. Manser, Ruben Perez-Carrasco

**Affiliations:** Department of Life Sciences, Imperial College London, South Kensington Campus, Imperial College London, London SW7 2AZ, UK

**Keywords:** Cell fate decision, Dynamical systems, Gene regulation, Landscape, Mathematical modelling, Tempo

## Abstract

Embryo development is a dynamic process governed by the regulation of timing and sequences of gene expression, which control the proper growth of the organism. Although many genetic programmes coordinating these sequences are common across species, the timescales of gene expression can vary significantly among different organisms. Currently, substantial experimental efforts are focused on identifying molecular mechanisms that control these temporal aspects. In contrast, the capacity of established mathematical models to incorporate tempo control while maintaining the same dynamical landscape remains less understood. Here, we address this gap by developing a mathematical framework that links the functionality of developmental programmes to the corresponding gene expression orbits (or landscapes). This unlocks the ability to find tempo differences as perturbations in the dynamical system that preserve its orbits. We demonstrate that this framework allows for the prediction of molecular mechanisms governing tempo, through both numerical and analytical methods. Our exploration includes two case studies: a generic network featuring coupled production and degradation, with a particular application to neural progenitor differentiation; and the repressilator. In the latter, we illustrate how altering the dimerisation rates of transcription factors can decouple the tempo from the shape of the resulting orbits. We conclude by highlighting how the identification of orthogonal molecular mechanisms for tempo control can inform the design of circuits with specific orbits and tempos.

## Teaser

**Highlighted Article:** This works presents a mathematical framework to study molecular mechanisms controlling the timing of gene expression during embryo development, enhancing our understanding and design of developmental and synthetic biological circuits.

## INTRODUCTION

Biological systems are intrinsically dynamic. Cells are constantly migrating, differentiating, dividing and dying; collectively, these activities underpin numerous biological processes. This dynamism is especially apparent during embryonic development, during which the precise coordination of cell differentiation plays a pivotal role in the orchestrated formation of distinct tissues constituting the anatomical structure of the organism (Polleux et al., 1997; Colombani et al., 2012; Tuazon and Mullins, 2015; McLaren and Steventon, 2021; Ebisuya and Briscoe, 2018). This coordination takes place across different chemo-mechanical scales and is locally encoded at the cellular level through genetic programmes. Also known as gene regulatory networks, these programmes confer the cell with the dynamical properties required for different cell differentiation processes (Delás and Briscoe, 2020; Reik, 2007). Notably, any inaccuracies in the timing of cellular dynamics can lead to critical errors that disrupt the healthy development of the embryo (Polleux et al., 1997; Colombani et al., 2012). Conversely, flexibility in the timing between tissues allows for the plasticity required for evolutionary traits to emerge. Given the crucial role of timing, a fundamental question arises in the field of developmental biology: how is the timing of cellular decisions precisely controlled?

One strategy to answer this question involves comparing homologous developmental processes across different species (McNamara, 2012), particularly during the phylotypic stage when embryos closely resemble each other (Duboule, 2003). These processes usually preserve the same function across species, manifested as the same sequential pattern of activation of gene expression. Recent research has shown that these developmental programmes, with evolutionarily conserved cis-regulatory architectures, manifest different species-specific timings (Halley, 2022; Lázaro et al., 2023; Linaro et al., 2019). These different timings can vary by an order of magnitude across distinct species, and can be observed in processes such as motor neuron differentiation (Rayon et al., 2020), the segmentation clock (Diaz-Cuadros et al., 2020; Matsuda et al., 2020), and corticogenesis (Iwata et al., 2023). During each one of these processes, the same genes are expressed in the same sequential manner, but at different speeds. Drawing an analogy from music, the species-specific execution speed of otherwise analogous genetic programmes has been termed the tempo of the system (Rayon et al., 2020; Diaz-Cuadros and Pourquié, 2023; Rayon, 2023; Simpson and Alberio, 2023).

Furthermore, the species-specific tempo in these processes can be inherently cell intrinsic, not requiring intercellular communication (Ebisuya and Briscoe, 2018; Busby and Steventon, 2021; Fuhrmann et al., 2020). This observation has resulted in an extensive search for the intracellular mechanisms governing tempo, with recent candidates focusing on specific molecular steps of gene regulation, such as protein stability, mRNA splicing, phosphorylation or ubiquitylation (Rayon et al., 2020; Matsuda et al., 2020; Blatnik et al., 2022; Sonnen, 2023; Iwata et al., 2023; Diaz-Cuadros et al., 2023). Contributing to this complexity, all these different mechanisms can affect each other, which, given the inherent non-linearity of gene regulation, impedes a straightforward mechanistic identification of tempo control.

The impact of non-linear feedback in gene regulation has been efficiently tackled in the past through mathematical modelling within the dynamical systems framework. The success of this strategy resides in its ability to connect molecular mechanisms with a general understanding of the compatible dynamics (Tomlin and Axelrod, 2007; Cooke and Zeeman, 1976; von Dassow et al., 2000; Dillon et al., 2003; Erban and Othmer, 2005). Yet the vast majority of the dynamical systems literature has been centred around steady-states, usually associated with available cell types, and very few studies have focused on the transient dynamics leading to those states (Tufcea and François, 2015; Zhu et al., 2017). This gap highlights the need for novel tools to comprehend the non-linear mechanisms governing tempo variations across species. Here, we devise a mathematical approach that connects the dynamic sequence of relative gene expression levels with the underlying biomolecular processes.

## A MATHEMATICAL FRAMEWORK FOR FUNCTION AND TEMPO

### Orbital equivalence: preserving the function of gene regulatory programmes

Before attempting to compare quantitatively the tempo between two species, it is necessary to ask whether they can actually be compared. Similarly, when we claim that the differentiation of two cells is the same except for their tempo, what do we mean by ‘the same’? In essence, we need to identify the differential characteristics of two temporal gene expression profiles that allow us to assert that they perform the same function but at a different tempo. Within the context of cell differentiation, one way to contain the function of a genetic programme is the conserved sequence of relative expression of different genes, independently of the speed at which this sequence is travelled. This definition is a useful proxy for biological function as it maintains many of the markers traditionally used to study embryonic development.

**Fig. 1.**
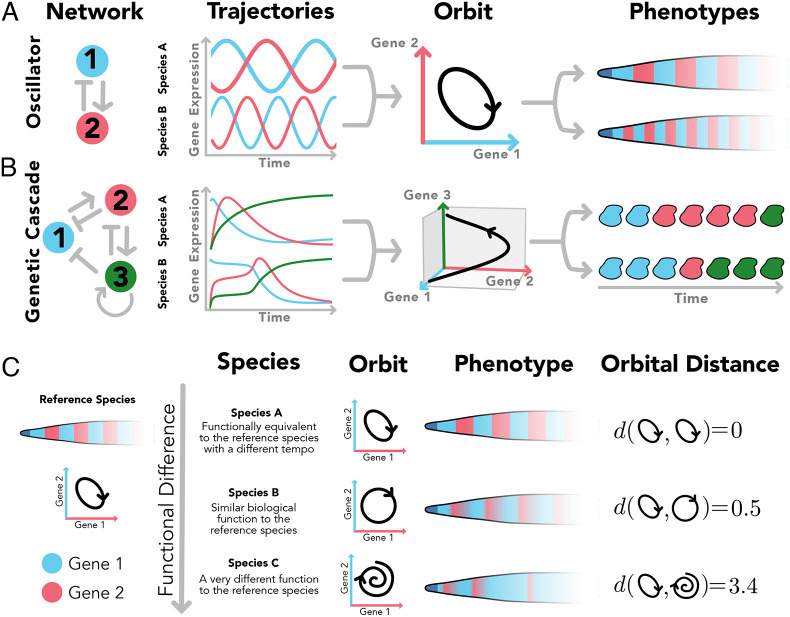
**Orbital distance allows comparison of the function of gene regulatory networks of two species.** (A,B) Two species (systems) that show the same relative sequences of gene expression in time will share the same orbit even though they might have different trajectories. This is translated in a different tempo while preserving the activity-function of the system. This is shown for an oscillator in charge of some spatial patterning (A), where the cellular states forming the pattern have been preserved by conserving the relative sequences of gene expression, and for more complex networks in charge of a cascade of gene activations (B), where the sequence of cell states is also preserved. (C) Distance between activity-functions for different species can be studied by comparing the orbits of the species defining an orbital distance, ***d***.

Adopting the jargon of dynamical systems to describe the situation in which two cells can have different gene expression trajectories, while still following the same orbits of gene expression, the orbits of the system can be visualised as the continuous sequence of cell states in the gene expression phase-space (Fig. 1A,B).

This identification of gene expression orbits with their core functional activity harmonises with the proposed concept of dynamical modularity proposed by Jaeger and Monk (2021): ‘A dynamical module or subsystem is defined by an elementary—distinguishable and quasi-autonomous—activity-function that corresponds to some dynamical regime within the broader dynamical repertoire of a complex system’. This way, the function of a system (or ‘dynamical module’) can be defined by ‘what the module does or, more precisely, by the particular kind of dynamics it generates’. It should be noted that other notions of function exist, such as the ‘use-function’, for which the function is determined by the downstream effects of the dynamical module, i.e. the effect that changes in the system have on the long-term development of the organism. We have decided against this definition in favour of activity-function, as we know from *in vitro* studies that tempo is cell intrinsic (unlike use-function), and because incorporating all the complexities of downstream effects would not be possible. Henceforth, here we will adopt Jaeger and Monk's ’activity-function’ definition when referring to ‘function’.

We can employ this definition to compare how close the functions of two different dynamical systems are by quantifying the similarity of their gene expression orbits (Fig. 1C). This strategy also reframes our exploration of tempo-controlling mechanisms: we can search for a suite of biochemical perturbations that sustain the integrity of a system's orbit. Thus, the effect of these biochemical perturbations can exclusively change the tempo. In addition, given the ability of mathematical models to incorporate different levels of biochemical detail in gene expression, we can use this framework to compare how different molecular mechanisms (e.g. post-translational modifications or promoter occupancy) affect the orbits of expression of given genes.

**Fig. 2.**
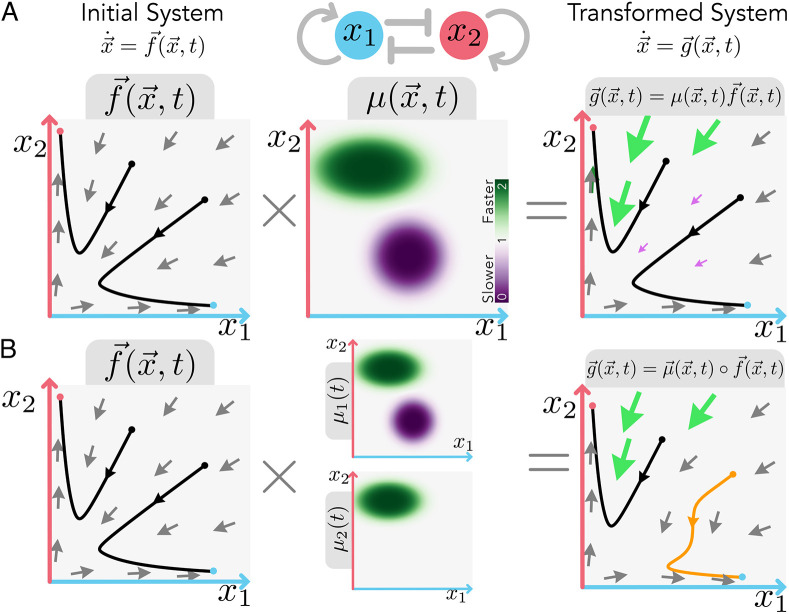
**Orbital equivalence dissociates dynamical landscape and tempo between two dynamical systems.** (A) A dynamical system 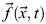 (left) for a given gene regulatory network [in this case, a bistable switch with two possible attracting states denoted by red and blue circles in the phase space (*x*_1_, *x*_2_). Two different orbits (black lines) are shown for two given initial conditions (black circles). The dynamical system can be transformed into a different system that preserves the same orbits 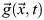 (right), by multiplying the initial system by a prefactor, 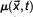 (centre), that preserves the dynamical landscape (direction of flow). The prefactor might vary arbitrarily in space and time, giving rise to zones of the gene expression landscape that are faster (green arrows and zones), or slower (purple arrows and zones), while keeping the same orientation of the evolution of the system 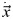 at any point 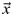. (B) If the prefactor is not the same for every gene (so that it is represented as a vector 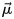 rather than a scalar 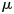), the resulting orbits may not be preserved. In this scenario, the system 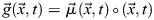 results from the element-wise multiplication (also known as Hadamard product) for each component 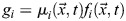. Prefactor heterogeneity among genes only impacts orbits in gene expression zones where the prefactors differ (depicted by the orange orbit). Parameters and functions used can be found in the supplementary Materials and Methods.

Genetic networks can be modelled in a variety of ways, such as with discrete or stochastic descriptions (Gillespie, 2000). For the purpose of this study, we will describe gene expression as a set of biochemical reactions that can be modelled as a system of ordinary differential equations (Briscoe, 2019; Milo et al., 2002; Exelby et al., 2021). In this description, the evolution of *N* different biochemical species in time 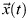 with 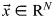 results in the following description of their rates of change:
(1)

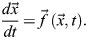
The functions 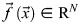 encapsulate the biochemical reactions describing the system. In general, to obtain the orbits of a genetic programme, numerical integration of Eqn 1 is required. However, we can identify whether two systems have the same orbits by directly inspecting their systems of equations. Specifically, when considering a second dynamical system given by 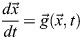 with 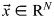, both systems share identical orbits if:
(2)

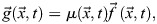
when 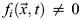. In other words, two systems have the same orbits when a scalar prefactor, 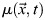, exists that scales the rates of change of the different biochemical species (Kuznetsov, 1998). When this occurs, it is said that both systems are orbitally equivalent. The prefactor 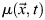 has a straightforward interpretation. It serves as the constant that scales the rate of change for each gene locally at any given specific gene expression state. This scaling relationship is shared among all the genes 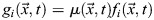 for genes *i*=1,..., *N*. This preserves the overall direction of the rate vector 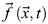 in the phase-space while keeping intact the resulting orbits (Fig. 2). In the context of dynamical landscapes, commonly used to understand differentiation trajectories (Sáez et al., 2022; Ferrell, 2012; Wang et al., 2011; Verd et al., 2014), the prefactor 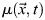 encodes all the possible changes that can be performed to the dynamical system that preserve intact the landscape (i.e. the direction of the flow).

Moreover, 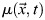 quantifies the relative tempo of a particular process given by its harmonic mean along the orbit of that process, 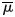, written as the ratio of line integrals (see supplementary Materials and Methods for a detailed derivation):
(3)

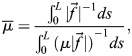
where *L* is the total length of the orbit in the state space. Note that orbital equivalence leaves plenty of freedom to the form of the prefactor 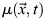, which can change arbitrarily in the gene expression space (Fig. 2A). Hence, under the lens of dynamical systems, it becomes apparent that tempo changes are not limited to global constant organism-specific tempos (i.e. allochrony), but more generally to changes in the local speed (dependent on the gene expression state, 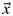) at which the same gene dynamical landscape is traversed (i.e. general heterochrony). One could assess how far from allochrony a particular system is by using a measure of the deviation of 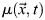 from the average 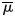 along an orbit, e.g. the distance 
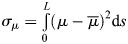
. The concept of continuous tempo changes along the orbit remains consistent with the musical analogy, whereby variations in speed occur within a piece without altering the sequence of notes played.

### Orbital distance: comparing the function of gene regulatory programmes

In general, it is not anticipated that two organisms will exhibit precisely identical orbits, as slight variations in orbits will maintain the overall function of the cell differentiation programme (Fig. 1C). Similarly, changes in biochemical rates are not expected to result in exact orbital equivalence (Eqn 2). Taken together, this highlights the requirement to define an orbital distance that measures the divergence between orbits in different systems.

Here, we will evaluate the distance of two orbits using the Fréchet distance, which captures the maximum deviation between two orbits, becoming zero when two systems are orbitally equivalent. The Fréchet distance between paths A and B can be intuitively understood as the following: the Fréchet distance is the minimum length of a leash required if a person were to walk along path A and their dog along path B, with both allowed to vary their speeds independently. A formal definition can be found in the supplementary Materials and Methods. Several alternative definitions of orbital distance are possible (Cleasby et al., 2019; Tao et al., 2021), each able to capture different aspects of the similarity between two different gene expression sequences (see supplementary Materials and Methods and Fig. S1 for more examples of possible measures). For example, a commonly used measure in biology is dynamical time warping (DTW). The main difference between Fréchet distance and DTW is that the latter is an aggregate method (accumulating differences along both orbits), whereas Fréchet is a bottleneck method, identifying the most extreme difference between two orbits. We focus on the former, because it prioritises penalties of local dramatic deformations of the orbit as opposed to a small orbit shift.

Ultimately, the choice of orbital distance needs to address how similar the functions of two systems are. This is not only required for two mathematical models to be compared, but also applies to the comparison of two experimental datasets of gene expression for which tempo is interrogated (regardless of whether the underlying gene regulatory network is known).

In addition, when investigating the role of different molecular mechanisms using the dynamical systems modelling framework, we can also attempt to define an analytical orbital distance using the definition of orbital equivalence (recalling Eqn 2). This can be done by searching for parameter modifications that can be written as a multiplicative prefactor in the dynamical system. In general, it is not expected that arbitrary perturbations will entail exact orbital equivalence. Instead, changes in biochemical parameters that preserve the function of the system will involve a gene-dependent prefactor (i.e. a vector 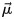 that is no longer the same scalar for each single gene) that is comparable across different genes. The new system follows the element-wise (or Hadamard) product 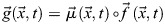, where 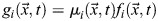. When the components of the prefactor are different from each other, the systems will not be orbitally equivalent (Fig. 2B). Hence, an orbital distance can be defined by quantifying the heterogeneity of the components of the prefactor vector 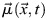. This can be achieved through measures such as the weighted variance:
(4)

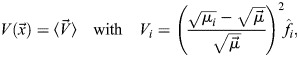
where 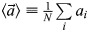 is the average taken over the components of a vector 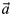. The weights 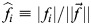 correspond to the normalised velocity vector's components (Eqn 1). These weights ensure that heterogeneity is accounted for in the molecular species that shape the system's orbit at certain points in the state space. For instance, a prefactor component *μ*_*j*_ different from the system's average 〈*μ*_*i*_〉 will not compromise orbital equivalence within regions of the state space where that gene expression is not changing, i.e. *f*_*j*_≃0. Finally, we want to evaluate the heterogeneity 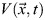 along the system's orbit. To compare with the results from the Fréchet distance, we can define an orbital prefactor heterogeneity distance as the maximum heterogeneity along the orbit:
(5)

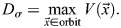
For two identical orbits, *D*_*σ*_=0, and *D*_*σ*_ grows as the prefactors become more dissimilar. Together with our measure of tempo (Eqn 3), we can interrogate how different mechanisms influence these factors by searching for mechanisms that minimise *D*_*σ*_, but the tempo is allowed to vary.

## APPLICATIONS

### Scaling production and degradation

**Fig. 3.**
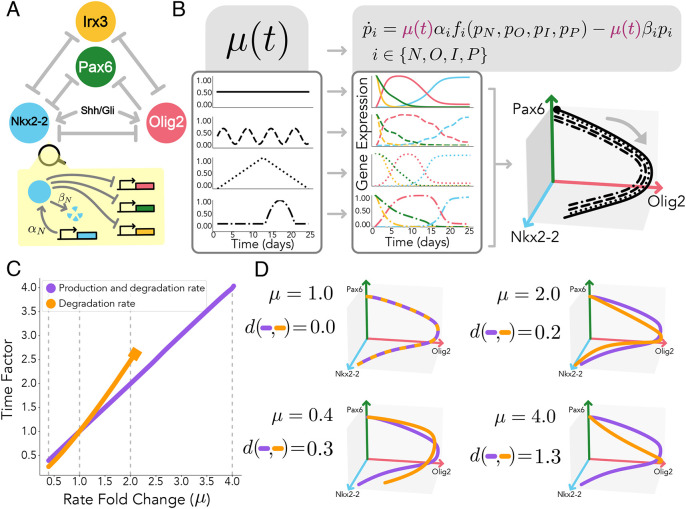
**Scaling of production and degradation in neural precursors can control differentiation tempo.** (A) The gene regulatory network in charge of ventral neural tube differentiation in vertebrates, together with a molecular schematic of the mechanisms included in the model. (B) Various arbitrary prefactors, *μ*(*t*) (indicated by different dashed lines in the left column), which scale the protein production and degradation, result in different trajectories of gene expression during V3 interneuron differentiation (centre) while keeping the same orbit (right plot, where the perfectly overlapping orbits have been shifted for visualisation purposes). This changes the tempo of the genetic cascade while keeping the relative expression and sequence of gene activation along the switch. (C) Rate fold change versus time factor for the two different strategies used to explore changes in tempo during V3 interneuron differentiation: change of the degradation rates (orange), and combined change of production and degradation rates (purple). Changes in degradation rates only cannot explain time factors for large changes in the degradation rates, failing to express Nkx2-2 beyond a critical value of degradation rates (orange square). (D) Example orbits for four different cases of rate fold change: *μ*=1.0, 0.4, 2.0 and 4.0 (dashed grey lines in C). As the rate fold change increases, changes in degradation rate (orange) perturb the orbit, increasing the orbital distance, *d*(). Large values of the degradation rate (*μ*=4.0) prohibit the progression to the Nkx2-2 state, resulting in a motor neuron expression profile (high Olig2). By contrast, rescaling production and degradation rates in tandem never deforms the orbit, and therefore any tempo change is possible. Parameters and functions used can be found in the supplementary Materials and Methods.

One immediate result from the orbital equivalence framework is that identical global rescaling of protein production and degradation rates of the transcription factors of a given gene regulatory network will preserve the orbit while controlling tempo. Interestingly, this rescaling does not have to be constant in time but can change arbitrarily in time (or even gene expression state). To illustrate this result, we analysed the dynamics of gene expression for a well-studied model of differentiation of neural progenitors in the ventral neural tube that includes the interaction of four genes (Fig. 3A) (Cohen et al., 2014; Rayon et al., 2020). Neural precursors initially express Pax6 and Irx3, and, when subjected to high Shh signal levels, undergo a transient gene expression cascade. This process involves the orderly activation/deactivation of genes within the network, culminating in the expression of Nkx2-2, which steers the precursors towards a V3 interneuron identity. We modified the model by controlling a prefactor, *μ*(*t*), multiplying the production and degradation rates (Fig. 3B). As expected, different temporal profiles of *μ*(*t*) yield different trajectories. Nevertheless, the orbits of gene expression are conserved, i.e. the sequence of relative expression of all the genes involved in the neuron differentiation process is unaltered, including the final expression state.

Hence, this rescaling provides a general mechanism to preserve the orbit of gene expression independently of changes in rates along the cell cycle or through external perturbations as long as production and degradation are rescaled in the same way. This result applies to any given regulatory network independently of the complexity of its topology, suggesting a robust mechanism to tune tempo of a regulatory system, allowing for evolutionary strategies.

Recent experimental work by Rayon et al. (2020) on neural precursor differentiation used the same model to demonstrate that protein degradation rate can control tempo. In Fig. 3C, we compare the time factor resulting from a modification of degradation rates only (as was done in the Rayon et al. study), versus a global rescaling of production and degradation. As expected, we observed that global rescaling affects the tempo linearly, and indefinitely, achieving any possible tempo. By contrast, modifying degradation rates alone restricts the range of achievable tempos, meaning that the Nkx2-2 state cannot be achieved when the change in degradation rate exceeds a twofold increase. This failure stems from the fact that isolated changes in protein degradation rate alone cannot maintain the shape of the orbit (Fig. 3D). In this case, it can even change the final steady-state of the trajectory. This phenomenon is further observed by an increase in orbital distance, which grows as the degradation rate changes. A significant jump in the orbital distance occurs when the Nkx2-2 state becomes unattainable, thereby altering the cell fate to a high Olig2 (motor neuron) fate.

Therefore, although changing degradation rates can explain the time factor difference of 2.5 between human and mouse reported by Rayon et al. (2020), it cannot immediately explain the greater differences present between other species. For example, zebrafish neural precursor differentiation lasts less than a day, whereas for humans it takes around two weeks (Rayon and Briscoe, 2021), a time factor of 10 or more. Supporting our predicted general mechanism of tempo control, recent experimental studies investigating the species-specific timing of the segmentation clock (Matsuda et al., 2020) have identified differences in protein stability and production rates across species. Although we cannot conclude that there are other molecular mechanisms at play allowing the extension of tempo beyond the limits allowed by changes in degradation rates, our framework proposes a straightforward molecular strategy.

### Case study: the repressilator

**Fig. 4.**
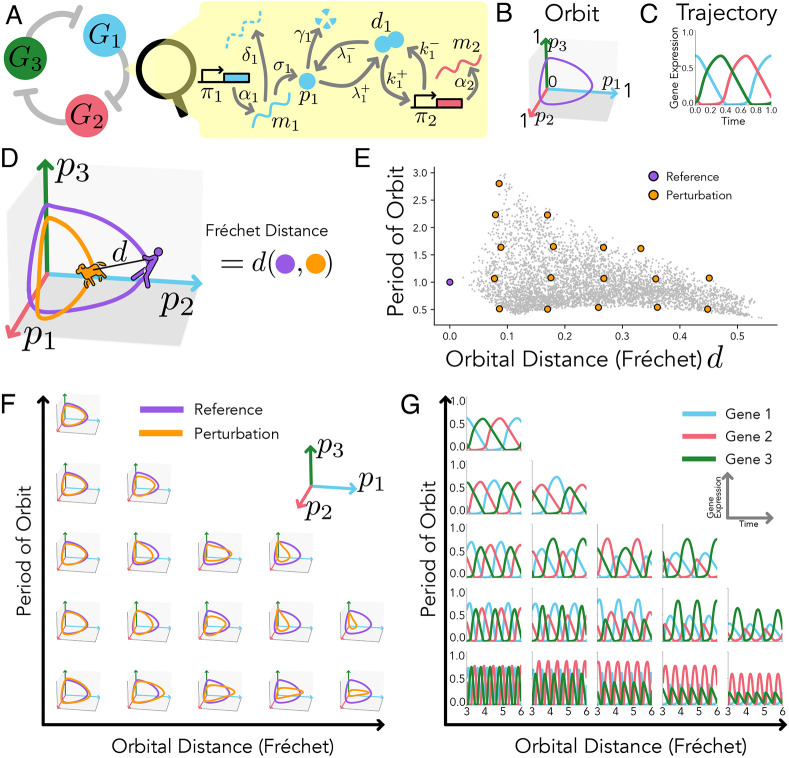
**Molecular perturbations can affect orbit and the tempo.** (A) A schematic of the repressilator, in which three genes repress each other in turn (left) with the intermediate biochemical steps considered (right). (B,C) The repressilator can give rise to stable oscillations of gene expression that result in closed orbits (B) and periodic trajectories (C). (D) The orbital distance between two different systems is measured with the Fréchet distance, a measure of similarity between two curves. The Fréchet distance can be intuitively understood as the shortest lead distance, ***d***_,_ needed for a person walking a dog, where the person walks along one curve, and the dog walks along the other, allowing both to move at independent speeds. (E) Resulting period and orbital distance of 5000 randomly selected perturbed systems (grey dots) with respect to a reference system (purple circle). Each parameter set, *r*_***i***,_ was obtained by perturbing the reference parameter set 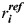 with a uniform fold change. A subset of these systems (orange circles) is then focused on in F to examine their orbits, and in G to examine their trajectories over time. The relative position of the subplots in F and G correspond to the relative position of the corresponding parameter set (orange circle) in E. (F) Orbits for selected perturbed parameter sets (orange circles in E), confirm that some parameter sets can keep the orbit shape while changing tempo (period of the orbit), whereas others change the orbit of the system while keeping the same period of oscillation. (G) Trajectories for the same selected parameter sets showing the perturbation effect in the temporal evolution of gene expression.

In general, arbitrary perturbations other than global scalings of production and degradation will not necessarily result in orbital equivalence. To showcase the applicability of the orbital equivalence framework in these scenarios, we implemented it within a well-known gene regulatory network, the repressilator (Fig. 4A). Despite not corresponding with a particular developmental biology example, it is a paradigmatic circuit capable of gene expression oscillations that offer the flexibility to incorporate different degrees of biochemical complexity (Perez-Carrasco et al., 2018; Elowitz and Leibler, 2000; Wu et al., 2017) and different tempos (Zhang et al., 2022). In addition, sustained oscillations result in closed orbits in the state-space (Fig. 4B,C), allowing for a clearer visualisation of the concept of orbital distance.

To explore the possibility of different post-transcriptional mechanisms, we employ the molecular description presented by Bennett et al. (2007), containing an explicit dimerisation and promoter occupancy (Fig. 4A). In particular, each one of the three genes composing the circuit has four distinct variables associated with it: mRNA (*m*_*i*_), protein (*p*_*i*_), protein dimer (*d*_*i*_), and promoter occupancy with two possible states with a probability of being active (*π*_*i*_). The evolution of these 12 variables is given by:
(6)

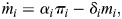

(7)

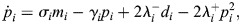

(8)

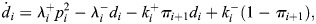

(9)

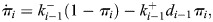
where *i*=(1, 2, 3) is a cyclic index running through the three genes. This description contains eight reactions for each gene, yielding a total of 24 different biochemical rates (Fig. 4A): transcription (*α*_*i*_), translation (*σ*_*i*_), degradation of mRNA (*δ*_*i*_), degradation of protein (*γ*_*i*_), reversible dimerisation (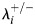), and reversible promoter binding of a dimer, which silences the transcription of the succeeding gene (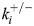).

This high-dimensional system can be reduced by leveraging the difference in timescales of the dynamics of the different biochemical species. Employing quasi-steady-state approximation (QSSA) methods (Bennett et al., 2007; Fenichel, 1979; Heineken et al., 1967; Goeke et al., 2017), we can simplify the system of equations by assuming that the change in time of total protein amount in any of its configurations (monomer, free dimer, or bound dimer) changes at a slower timescale than the relative differences between any of these possible configurations. Under this assumption, we can reduce the system to only two sets of equations for each gene (see supplementary Materials and Methods for more details):

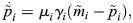

(10)

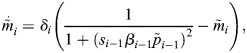

with
(11)

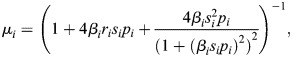
where we have introduced three new parameters that control the system dynamics: 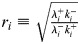, 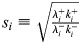, 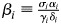. In particular, *r*_*i*_ and *s*_*i*_ control the directionality and relative magnitude of the reversible intermediate reactions that connect the monomeric protein and the bound promoter. Given that we are interested in the overall shape of the orbit regardless of the absolute number of molecules, without any loss of generality we have also rewritten the dynamics in terms of the non-dimensionalised mRNA and protein expression (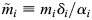 and 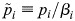). Note that this choice impacts the calculation of orbital distances, capturing changes in gene expression relative to maximum levels. Different normalisation methods are also possible, reflecting other biological functions of levels of gene expression. Finally, we can assume that mRNA dynamics reach equilibrium fast with respect to the value of 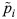. Applying QSSA to mRNA we can simplify the system further (see supplementary Materials and Methods):
(12)

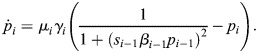
It can be seen that the effect of the intermediate protein mechanisms is summarised as a prefactor, *μ*_*i*_. Comparing this result with Eqn 2, we can identify *μ*_*i*_ as the prefactor in charge of orbital equivalence. In particular, the only biochemical parameters that appear exclusively in the prefactor (see Eqns 11 and 12) are *r*_*i*_, controlling the reaction flux of free dimer, suggesting a route to control tempo. Because the three genes have different prefactors *μ*_*i*_(*p*_*i*_), which depend on the parameters and gene expression, we need to employ the different tools of orbital distance to assess the limits and characteristics of tempo control.

To explore the dynamical properties available through changes in the directionality of reactions towards the dimer state *r*_*i*_, we undertook a parameter search for different perturbations of the parameters *r*_*i*_ with respect to an arbitrarily chosen reference system. Analysis of the resulting oscillatory systems shows high variability in the possible oscillatory behaviours (Fig. 4D,E). To visualise the results of this search, we can pick out a subset of systems from Fig. 4E (highlighted in orange) and display their orbits (Fig. 4F) and trajectories (Fig. 4G) in a grid reflecting their relative position in Fig. 4E. For both Fig. 4F and G, rows correspond to systems with the same period (tempo), and columns correspond to systems with the same orbital distance to the reference system, which increases as you go from left to right. The resulting orbits not only exhibit a large variability of shapes (Fig. 4F), but there is also variability in the periods of the orbits (Fig. 4G). Most interestingly, there are many cases in which the period of the orbit can be perturbed without changing the orbit of the system (see the left-most column of Fig. 4F,G). Similarly, there are cases in which different orbits are obtained while preserving the period of the oscillations, suggesting that there are orthogonal molecular strategies to change orbit shape and tempo.

### Identifying molecular mechanisms of orbital distance and tempo

**Fig. 5.**
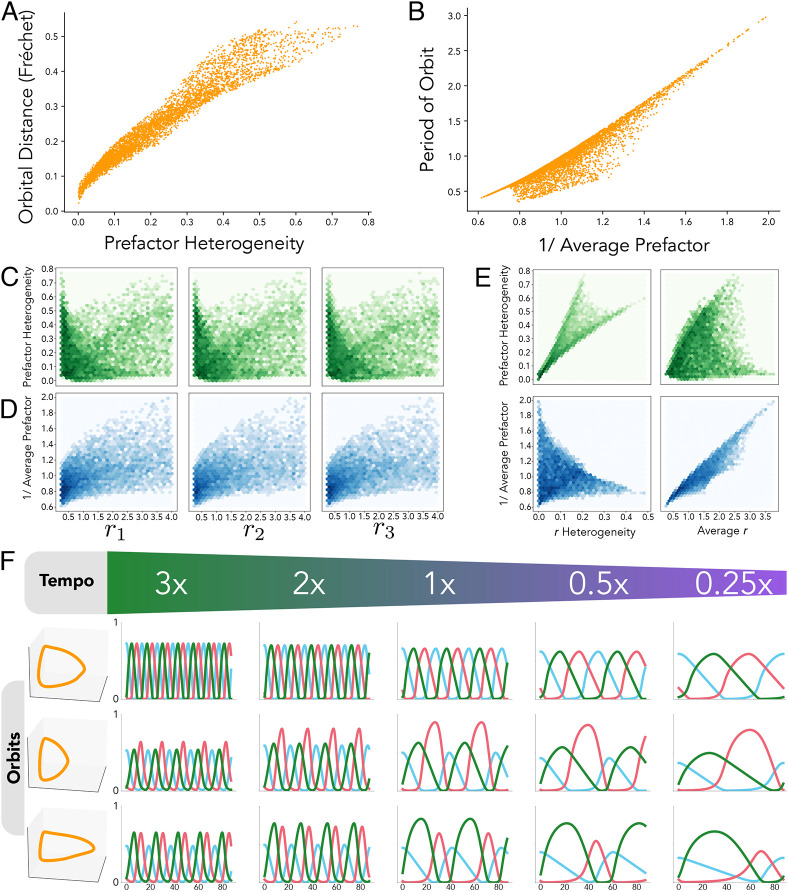
**Identification of tempo mechanism reveals molecular strategies of tempo control.** (A) Prefactor heterogeneity (Eqn 4) for the sampled points in Fig. 4 serves as a good predictor for orbital distance (calculated as the Fréchet distance to the reference orbit), allowing us to predict whether a parameter set will preserve the orbit without numerical integration. (B) Fold change of the average prefactor with respect to the reference system, calculated as 
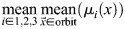
, gives a good prediction of the fold change in the orbit's period. (C,D) Individual values of *r*_***i***_ are not enough to predict the prefactor heterogeneity (green), or the average prefactor (blue). (E) The aggregate measurements ***r*** heterogeneity and 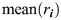 are good predictors of the prefactor heterogeneity and the average prefactor, respectively. (F) For three different chosen orbits (rows), five different molecular perturbations (columns) are designed that result in a system that preserves the orbit while changing the tempo. The parameter sets were designed by changing the mean value of *r*_***i***_ while keeping their heterogeneity (see supplementary Materials and Methods).

Observing the variability of behaviours accessible by changes in *r*_*i*_, we investigated whether we could predict the orbital distance and resulting period of the perturbed systems obtained in our parameter search by analytical inspection of the prefactors *μ*_*i*_ (Eqn 11). Using our analytical definition of prefactor heterogeneity *D*_*σ*_ (as outlined in Eqn 4 and Eqn 5) and comparing it with the orbital distance calculated by numerical integrations of the perturbed systems, we observed a linear relationship between the two (Fig. 5A). This relationship reveals an analytical means to predict the magnitude of the changes in the orbit's shape induced by a perturbation in the repressilator, without needing to integrate the perturbed ordinary differential equations.

Similarly, we investigated whether the period of oscillations in the perturbed system could be predicted by analysing only the prefactor of the new system. In this analysis, we found that the average prefactor serves as a reliable predictor of oscillation periods (Fig. 5B). This finding offers a method for forecasting the resulting oscillatory period without the numerical necessity of integrating the ordinary differential equations of the perturbed system.

Although the *μ*_*i*_ prefactors are good indicators of the effect of a perturbation in the system, they come with two main disadvantages. The prefactor needs to be evaluated along the orbit of the reference system (Eqn 5), and the prefactor is not immediately linked with the molecular mechanism in charge of orbital distance and tempo. Hence, we investigated whether we can identify molecular mechanisms of tempo and orbit shape control with definitions that link directly to the combined rates towards dimerisation *r*_*i*_. Initial inspection of the role that individual rates have on the prefactor reveals that, although there is some correlation between each *r*_*i*_ and the resulting prefactor heterogeneity and average prefactor, there is not a strong predictive relationship (Fig. 5C,D). These results suggest that the effect that changes of prefactor have on the orbit originate from a combination of perturbations in the ensemble of rates included in each *r*_*i*_. In particular, we identified that the average of the composed dimerisation rates can be used as a good predictor of the average prefactors, and consequently the period of the resulting orbit (Fig. 5E, Fig. S2B). However, we also find that the heterogeneity of *r*_*i*_ allows us to predict the prefactor heterogeneity, resulting in a predictor of the orbital distance (Fig. 5E, Fig. S2C). Therefore, our framework has allowed us to find underlying mechanisms (*r*_1_, *r*_2_ and *r*_3_) that affect orbital distance. Unlike the orbital distance, or prefactor analysis (e.g. *D*_*σ*_), calculating *r*_*i*_ heterogeneity and average does not require any knowledge of the orbit of the reference system. Instead, it provides direct molecular mechanistic insight into the effects of post-translational rates on trajectories.

These results provide two orthogonal molecular mechanisms to control orbit shape and tempo. Hence, this allows us to use these mechanisms to design sets of parameters that will preserve the orbit of a system while exclusively changing the tempo of the system, independently of the reference system. We can achieve this by tuning the average *r*_*i*_ while fixing the heterogeneity in the prefactor. Implementing this strategy, we successfully gained the capability to fine-tune the tempo for orbits of various shapes (Fig. 5F), resulting in achievable speed differences spanning a full order of magnitude.

## DISCUSSION

We have developed a mathematical framework using dynamical systems that can be used for investigating molecular mechanisms in charge of inter-species differences in developmental tempo. This framework is based on the notion of orbital distance, assigning a dynamical function to a system based on a defined progression of relative gene expression states. We have shown how we can explore this idea in two parallel ways: (1) by comparing orbits directly from gene expression data, and (2) by providing an analytical toolkit connecting ordinary differential equation representations with developmental tempo.

The versatility of the techniques introduced means that they can be applied to many different genetic programmes in development. The similarity measures based on gene expression data can be immediately used to compare function between organisms regardless of the underlying gene regulatory network. Although this paper employs the Fréchet distance for this comparison, many other possible distances are possible (see supplementary Materials and Methods), reflecting different definitions of function in embryogenesis. Similarly, we focus our definition of ‘function’ as the activity-function resulting from the orbit of a dynamical system, but identical orbits with different tempo can still entail different phenotypes (e.g. patterns of different width in the oscillatory example of Fig. 1), requiring the concept of function to be revisited when employing the framework in different systems.

The mathematical framework becomes very useful when contrasted with more traditional representations of the differentiation process as trajectories in a Waddingtonian landscape. Recent advances in this field have focused on reconstructing these landscapes from experimental data, usually fixing the location and change of steady states and basins of attraction of the landscape (Sáez et al., 2022; Verd et al., 2014). Here, we show that there are infinite available transformations of the dynamical system that preserve the same identical landscape. This transformation can be achieved through a prefactor of the dynamics (Fig. 2A). Overall, this reveals two compatible formalisms that separate the geometry and the tempo of a dynamical system's landscape. This not only allows us to understand the underlying mechanisms of tempo control, but also informs how we interpret and find landscape representations of a particular differentiation process.

Although our concept of orbital equivalence focuses on a preservation of the direction of the flow along a typical differentiation trajectory, further research should be done to include the robustness of these orbits under noise, delays, or changes in the initial conditions. In this latter case, the concept of orbital equivalence is closely related to the concept of homeorhesis (Verd et al., 2014; Young et al., 2017; Tufcea and François, 2015), whereby the relevant orbit of the system is described by the slow manifold of the dynamical system, buffering the effect that changes in the initial conditions have on the resulting orbit.

One of the main features of this study is showcasing that tempo can be controlled through intermediate reaction steps during the regulatory process. Previous experimental studies have shown that inter-species developmental tempo depends heavily on specific biochemical parameters (Matsuda et al., 2020; Rayon et al., 2020; Lázaro et al., 2023; Filina et al., 2022). Thus, the tools developed could be employed to investigate such systems to understand the impact and limits that different parameter perturbations will have on the resulting trajectories of gene expression. In order to do so, quasi-steady-state approximations need to be developed that can capture the impact that metabolic and other biochemical processes have on the overall change of gene expression in the state-space. In our case, we identified dimerisation rates as a pathway to control tempo; nevertheless, we anticipate that other intermediate steps may have similar temporal implications.

All in all, this framework not only shows a route to quantitatively connect molecular mechanisms with tempo in development, but it can also be extended to other areas. For instance, in an evolutionary context, the same tools can be used to identify possible molecular routes to change tempo while preserving the function of a system, hence identifying evolutionary strategies that showcase the molecular requirements at different tempos. Similarly, these strategies can be employed directly in synthetic circuit design, with the aim of building circuits that allow for tempo control of specific functions, or even controlling the tempo of *in vitro* cell differentiation.

## Supplementary Material



10.1242/develop.202950_sup1 Supplementary information
