# Supplementary material for: A mathematical framework for measuring and tuning tempo in developmental gene regulatory networks
Source: Development. 2024 Jun 24;151(12):dev202950. doi: 10.1242/dev.202950 (PMC11234385; doi:10.1242/dev.202950)
Supplement: Supplementary information [file develop-151-202950-s1.pdf]

Supplementary Materials and Methods

Tempo harmonic mean derivation

Given a dynamical system $\frac{d\vec{x}}{dt} = \vec{f}(\vec{x})$, the time T_f it takes to travel a certain orbit $\mathcal{C}$ of arclength L, follows

$$T_f = \int_0^{T_f} \mathrm{d}t = \int_{\mathcal{C}} \left|\frac{\mathrm{d}t}{\mathrm{d}s}\right| \mathrm{d}s = \int_0^L |\vec{f}|^{-1} \mathrm{d}s. \tag{S.1}$$

A second system orbitally equivalent to the first $\frac{d\vec{x}}{dt} = \vec{g}(\vec{x}, t) = \mu(\vec{x})\vec{f}(\vec{x})$ will take a time T_g following the same orbit $\mathcal{C}$

$$T_g = \int_0^{T_g} \mathrm{d}t = \int_0^L |\vec{g}|^{-1} \mathrm{d}s = \int_0^L |\mu\vec{f}|^{-1} \mathrm{d}s. \tag{S.2}$$

The relative tempo $\bar{\mu}$ between two orbits is the ratio of these two times

$$\bar{\mu} = \frac{T_f}{T_g} = \frac{\int_0^L |\vec{f}|^{-1} \mathrm{d}s}{\int_0^L (\mu|\vec{f}|)^{-1} \mathrm{d}s}. \tag{S.3}$$

Alternative definitions of orbital distance

In this manuscript, we have employed the Fréchet distance to measure the discrepancy between two orbits. Given two curves A and B. Then for any reparameterisation α and β of each curve, and the Euclidean distance $|\cdot|$, the Fréchet distance $F(A, B)$ is defined as

$$F(A, B) = \inf_{\alpha,\beta} \max_t |\alpha(t) - \beta(t)|$$

However, the literature offers a variety of alternative metrics for comparing curves. Some of these include Dynamic Time Warping (DTW), the Hausdorff Distance (HD), and the Distance Difference Matrix (DFD) [1] (Fig. S.1 A). These measures typically involve traversing both curves simultaneously and computing a distance metric during this process. For example, a common way of differentiating between curve distance methods is by classifying them as either bottleneck or aggregate methods. A bottleneck method is determined by finding the maximum point of difference between two curves. Meanwhile, an aggregate method calculates the distance as an average value along the entire length of both curves.

Another important consideration in selecting a distance metric is whether the comparison should be conducted by navigating both curves in a fixed sequential order (order dependent) or whether backtracking is permitted (order independent). This means that, when evaluating various orbits for their proximity to a reference orbit and arranging them in order, distinct curve distance measures may yield different rankings for the orbits (see Fig. S.1 C). Nevertheless, despite these differences, we observe similar results when using Fréchet, Hausdorff and DTW to compare against analytical measures of orbital distance through the prefactor (Fig. S.1 B) in that all give a monotonically increasing relationship.

2D Gene Regulation Equations

The system used to generate the bistable switch landscape of Fig. 2 in the main text is the following:

$$\begin{aligned} \dot{x} &= \mu(x, y)\left(\frac{1}{1 + 0.118\,(1 + 33y)^2} - x\right) \\ \dot{y} &= \mu(x, y)\left(\frac{1}{1 + 1.75 \times 10^{-4}\,(1 + 1000x)^2} - y\right). \end{aligned} \tag{S.4}$$

The scalar prefactor $\mu(\vec{x})$ for Fig. 2A is based on the two ellipses

$$\begin{aligned} c_1 &: 0.25 = e_1(x, y) = 2(x - 0.3)^2 + (y - 0.75)^2 \quad \text{and} \\ c_2 &: 0.3 = e_2(x, y) = (x - 0.5)^2 + (y - 0.3)^2, \end{aligned} \tag{S.5}$$

Development • Supplementary information

where c_1 determines the zone with increased tempo (green area in Fig. 2), and c_2 the area where dynamics slow down (purple area in Fig. 2). Inside ellipse c_1 the tempo is increased following the prefactor,

$$\mu(x,y) = 1 + \exp\left(-10^{-4}(e_1(x,y))^2\right), \tag{S.6}$$

while inside ellipse c_2 the tempo is decreased following the prefactor

$$\mu(x,y) = 1 - \exp\left(-1.25 \times 10^{-3}(e_2(x,y))^2\right). \tag{S.7}$$

The prefactor is kept intact ($\mu(x,y) = 1$) outside of both ellipses. For the vector prefactor $\vec{\mu}(x,y) = (\mu_1(t), \mu_2(t))$ in Fig. 2B, $\mu_1(x,y)$ is the same as the scalar μ above. However, $\mu_2(t)$ does not include the slow-down zone c_2.

Neural progenitor equations and parameters

The equations for the neural progenitor used in section 3.1 and Fig. 3 are based on the model in [2], under quasi-steady state approximation of the mRNA.

$$\dot{p}_i = \alpha f_i(\cdot) - \beta p_i$$

where

$$\begin{aligned}
f_N(p_O, p_I, p_P) &= \frac{K_{LN}L(1 + cK_{GN}G)}{K_{LN}L(1 + cK_{GN}G) + (1 + K_{GN})(1 + K_{ON}p_O)^2(1 + K_{IN}p_I)^2(1 + K_{PN}p_P)^2} \\
f_O(p_N, p_I) &= \frac{K_{LO}L(1 + cK_{GO}G)}{K_{LO}L(1 + cK_{GO}G) + (1 + K_{GO})(1 + K_{NO}p_N)^2(1 + K_{IO}p_I)^2} \\
f_I(p_N, p_O) &= \frac{K_{LI}L}{K_{LI}L + (1 + K_{OI}p_O)^2(1 + K_{NI}p_N)^2} \\
f_P(p_N, p_O) &= \frac{K_{LP}L}{K_{LP}L + (1 + K_{OP}p_O)^2(1 + K_{NP}p_N)^2}
\end{aligned}$$

Parameter	Value
K_{GO}	18.0 a.u.
K_{GN}	37.3 a.u.
K_{LN}	27.4 a.u.
K_{LO}	47.8 a.u.
K_{LP}	4.80 a.u.
K_{LI}	23.4 a.u.
K_{NP}	26.7 a.u.
K_{OP}	1.90 a.u.
K_{NO}	60.6 a.u.
K_{ON}	27.1 a.u.
K_{PN}	4.80 a.u.
K_{OI}	58.8 a.u.
K_{NI}	76.2 a.u.
K_{IO}	28.4 a.u.
K_{IN}	47.1 a.u.
c	10
α	0.089 h^{-1}
β	0.089 h^{-1}
L	0.8 a.u

Table S1. Parameters for the neural precursors.

The parameters used are summarised in Table 1, and correspond with the ones reported in [2]. We set the Gli signalling levels to 0.9, corresponding to a 15% dorso-ventral position across the neural tube with respect to the floor plate, in the v3 domain.

Development • Supplementary information

Derivation of the reduced dimension repressilator

In order to reduce the dimensionality of the full repressilator system (Eqs. (6)-(9) in the main text), we followed the same quasi-steady state approximation (QSSA) introduced by Bennet et al. [3]. The initial set of equations is

$$\begin{aligned}
\dot{m}_i &= \alpha_i \pi_i - \delta_i m_i, && \text{(S.8)}\\
\dot{p}_i &= \sigma_i m_i - \gamma_i p_i + 2\lambda_i^- d_i - 2\lambda_i^+ p_i^2, && \text{(S.9)}\\
\dot{d}_i &= \lambda_i^+ p_i^2 - \lambda_i^- d_i - k_i^+ \pi_{i+1} d_i + k_i^- (1 - \pi_{i+1}), && \text{(S.10)}\\
\dot{\pi}_i &= k_{i-1}^- (1 - \pi_i) - k_{i-1}^+ d_{i-1} \pi_i. && \text{(S.11)}
\end{aligned}$$

The meaning of the parameters are detailed in the main text, and summarized in Fig. 4A. Following QSSA, we identified that the molecular reactions changing the state of each individual protein (monomer p_i, free dimer d_i, or bound dimer through the promoter occupancy π_i) can be assumed to be faster than the change of total protein in any form.

Therefore, p_i, d_i and π_i equilibrate faster than the other species, so we can assume that equations (S.9), (S.10) and (S.11) are at equilibrium. Setting $\dot{d}_i$ to zero, equation (S.10) gives us

$$\lambda_i^- d_i - \lambda_i^+ p_i^2 = k_i^- (1 - \pi_{i+1}) - k_i^+ d_i \pi_{i+1}$$

The RHS of this equation is the RHS of equation (S.11) for $i \to i+1$. Since $\dot{\pi}_{i+1}$ is at equilibrium, we have

$$\begin{aligned}
\lambda_i^- d_i - \lambda_i^+ p_i^2 &= k_i^- (1 - \pi_{i+1}) - k_i^+ d_i \\
&= \dot{\pi}_{i+1} \\
&= 0.
\end{aligned} \tag{S.12}$$

Therefore, after rearrangement,

$$d_i = \frac{\lambda_i^+}{\lambda_i^-} p_i^2. \tag{S.13}$$

Substituting this into equation (S.11), given that $\dot{\pi}_{i+1} = 0$ gives us

$$\begin{aligned}
0 &= \dot{\pi}_i \\
&= k_{i-1}^- (1 - \pi_i) - k_{i-1}^+ d_{i-1} \pi_i \\
&= k_{i-1}^- (1 - \pi_i) - k_{i-1}^+ \frac{\lambda_{i-1}^+}{\lambda_{i-1}^-} p_{i-1}^2 \pi_i
\end{aligned} \tag{S.14}$$

And so, after rearrangement

$$\pi_i = (1 + \frac{k_{+,i-1}}{k_{-,i-1}} \frac{\lambda_{+,i-1}}{\lambda_{-,i-1}} p_{i-1}^2)^{-1}. \tag{S.15}$$

Setting $k_i \equiv \frac{k_{+,i}}{k_{-,i}}$, $\lambda_i \equiv \frac{\lambda_{+,i}}{\lambda_{-,i}}$, we have

$$d_i = \lambda_i p_i^2, \tag{S.16}$$

$$\pi_i = (1 + k_{i-1} \lambda_{i-1} p_{i-1}^2)^{-1}. \tag{S.17}$$

For usual QSSA, at this point we would substitute these equilibrium values into equations (S.9) and (S.8), giving us a 6-species system. However, as Bennet points out [3], this treats p_i as a solely 'slow' variable, even though it depends on dimerisation, a fast reaction. Instead, we define a new variable n_i, which is a truly 'slow' variable. The variable n_i represents the total concentration of protein i in any form, i.e. monomers, dimers, and those bound to regulatory sites. So,

$$\begin{aligned}
n_i &= p_i + 2d_i + (1 - \pi_{i+1}) \\
&= p_i + 2\lambda_i p_i^2 + k_i \lambda_i p_i^2 (1 + k_i \lambda_i p_i^2)^{-1}
\end{aligned} \tag{S.18}$$

Development • Supplementary information

Therefore,

$$\begin{aligned}\dot{n_i} &= \dot{p_i}\frac{\partial n_i}{\partial p_i} \\ &= \frac{\dot{p_i}}{\mu_i(p_i)} \qquad \text{with} \quad \mu_i(p_i) \coloneqq 1/\frac{\partial n_i}{\partial p_i}.\end{aligned} \tag{S.19}$$

Then

$$\mu_i(x) = \left(\frac{\partial n_i}{\partial p_i}\right)^{-1} = \left(1 + 4\lambda_i p + \frac{2k_i\lambda_i p}{(1+k_i\lambda_i p^2)^2}\right)^{-1}$$

In addition, from our definition of n_i as the total concentration of protein i in any form, we know that the only reactions that can change n_i are protein degradation and translation. so:

$$\dot{n_i} = \sigma_i m_i - \gamma_i p_i$$

And so (substituting into Eq. S.19)

$$\frac{\dot{p_i}}{\mu_i(p_i)} = \sigma_i m_i - \gamma_i p_i$$

Together this gives us the reduced system:

$$\begin{aligned}\dot{p_i} &= \mu_i(p_i)(\sigma_i m_i - \gamma_i p_i) \\ \dot{m_i} &= \frac{\alpha_i}{1 + k_{i-1}\lambda_{i-1}p_{i-1}^2} - \delta_i m_i\end{aligned}$$

Where

$$\mu_i(p) = \left(1 + 4\lambda_i p + \frac{2k_i\lambda_i p}{(1+k_i\lambda_i p^2)^2}\right)^{-1}$$

We can rewrite these equations in terms of the non-dimensional variables $\tilde{p}_i = \frac{p_i}{p_i^*}$ and $\tilde{m}_i = \frac{m_i}{m_i^*}$, where $p_i^* = \frac{\sigma_i\alpha_i}{\delta_i\gamma_i}$ is the maximum value of p_i in absence of repression (i.e. set $k_1^+ = k_2^+ = k_3^+ = 0$), and $m_i^* = \frac{\alpha_i}{\delta_i}$ is the maximum value of m_i in the absence of repression. We also let $r_i \equiv \sqrt{\frac{\lambda_i}{k_i}}$, $s_i \equiv \sqrt{\lambda_i k_i}$, $\beta_i \equiv \frac{\sigma_i\alpha_i}{\delta_i\gamma_i}$.

Without loss of generality, we can drop the tildes. These changes give us the following system:

$$\begin{aligned}\dot{p_i} &= \mu_i\gamma_i(m_i - p_i) \\ \dot{m_i} &= \delta_i\left(\frac{1}{1+(s_{i-1}\beta_{i-1}p_{i-1})^2} - m_i\right)\end{aligned} \tag{S.20}$$

Where

$$\mu_i = \left(1 + 4\beta_i r_i s_i x_i + \frac{4\beta_i s_i^2 x_i}{(1+(\beta_i s_i x_i)^2)^2}\right)^{-1}$$

and $r_i \equiv \sqrt{\frac{\lambda_i}{k_i}} = \sqrt{\frac{\lambda_{+,i}k_{-,i}}{\lambda_{-,i}k_{+,i}}}$, $s_i \equiv \sqrt{\lambda_i k_i} = \sqrt{\frac{\lambda_{+,i}k_{+,i}}{\lambda_{-,i}k_{-,i}}}$, $\beta_i \equiv \frac{\sigma_i\alpha_i}{\delta_i\gamma_i}$

Parameter search details

The parameter search was performed by generating 5000 sets of parameters perturbing a reference parameter system. The parameters for the reference system $r_i = r_i^{\text{ref}}$ can be found in Table 1. The perturbations from the parameters r_i are expressed as $r_i = r_i^{\text{ref}}e^{\zeta_i}$, where ζ is a uniformly distributed random variable $\zeta_i \sim \text{Uniform}(-\log 4, \log 4)$. Each set of randomly picked parameters will correspond to a system (which can be thought of as the GRN for a simulated new species). We removed parameter sets that do not give oscillations, as these parameters perturb the system so much that the orbit is destroyed. The values of r_1, r_2 and r_3 respectively of the perturbed systems in Fig. 5F in the main text are shown in Table 2.

Table S2. Parameters for the repressilator used in the parameter search.

Symbol	Meaning	Value
α_i	Transcription rate	20
σ_i	Translation rate	10
γ_i	Protein degradation rate	1
δ_i	mRNA degradation rate	3
λ_i^+	Protein dimerization rate	10
λ_i^-	Dimer dissociation rate	10
k_i^+	Binding of dimer to promoter rate	10
k_i^-	Dissociation of dimer and promoter rate	10

Table S3. System parameters for Fig. 5F in the main text.

Tempo	3x	2x	1x	0.5x	0.25x
Orbit 1	0.33, 0.33, 0.33	0.50, 0.50, 0.50	1.00, 1.00, 1.00	2.00, 2.00, 2.00	4.00, 4.00, 4.00
Orbit 2	0.06, 0.01, 0.06	0.13, 0.03, 0.13	0.50, 0.10, 0.50	1.00, 0.20, 1.00	2.00, 0.40, 2.00
Orbit 3	0.13, 0.03, 0.03	0.25, 0.05, 0.05	1.00, 0.20, 0.20	2.00, 0.40, 0.40	4.00, 0.80, 0.80

Heterogeneity of rate r_i

The heterogeneity η of the perturbed rates r_i with respect to a reference system r_i^{ref} was calculated by quantifying the deviation of the components $r_i^* = r_i/r_i^{ref}$ with respect to the homogeneous case $\vec{r^*} = (1, 1, 1)$. While there are different choices for this comparison, for the case of the repressilator, we found that the best correlation against prefactor heterogeneity was achieved through the angle θ between the homogeneous case $(1, 1, 1)$ and the vector $(\sqrt{r_1}, \sqrt{r_2}, \sqrt{r_3})$.

$$\eta = \tan^2\theta = \frac{1}{\cos^2\theta} - 1 = 3\frac{r_1^* + r_2^* + r_3^*}{\left(\sqrt{r_1^*} + \sqrt{r_2^*} + \sqrt{r_3^*}\right)^2} - 1 \quad (S.21)$$

Hence, different deviations from the homogeneous case can be visualised as cones around the vector (1,1,1) (see Fig. S.2 A)

References

[1] Fabian Schuhmann, Leonie Ryvkin, James D. McLaren, Luca Gerhards, and Ilia A. Solov'yov. Across atoms to crossing continents: application of similarity measures to biological location data. *bioRxiv*, June 2022.

[2] Teresa Rayon, Despina Stamataki, Ruben Perez-Carrasco, Lorena Garcia-Perez, Christopher Barrington, Manuela Melchionda, Katherine Exelby, Jorge Lazaro, Victor L. J. Tybulewicz, Elizabeth M. C. Fisher, and James Briscoe. Species-specific pace of development is associated with differences in protein stability. *Science*, 369(6510), September 2020.

[3] Matthew R. Bennett, Dmitri Volfson, Lev Tsimring, and Jeff Hasty. Transient dynamics of genetic regulatory networks. *Biophysical Journal*, 92(10):3501–3512, May 2007.

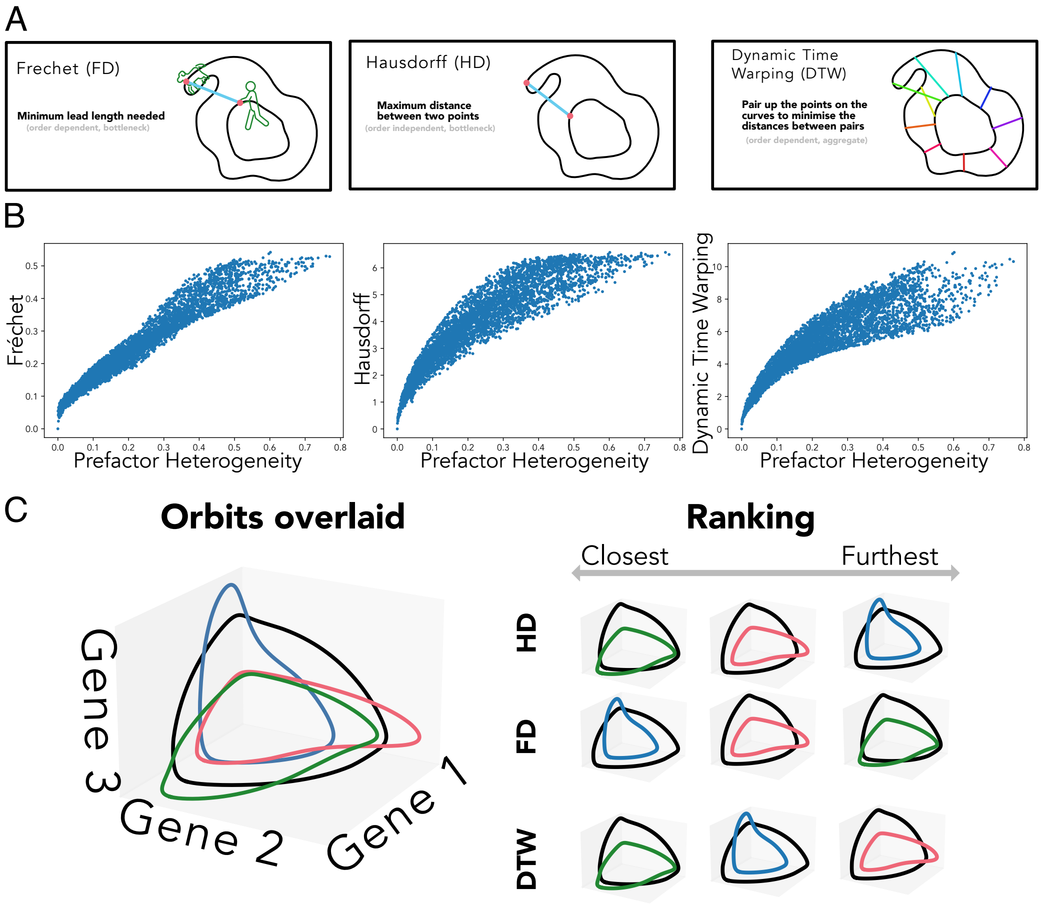

Fig. S1. A) Schematic comparing three different similarity measures. **B)** Comparisons of Fréchet, Hausdorff and dynamic time warping vs prefactor heterogeneity for the same perturbation set from Fig. 4C) Three different orbits in green, blue and pink are compared to a reference orbit (black curve). Different distance definitions rank the orbits in a different order.

Development • Supplementary information

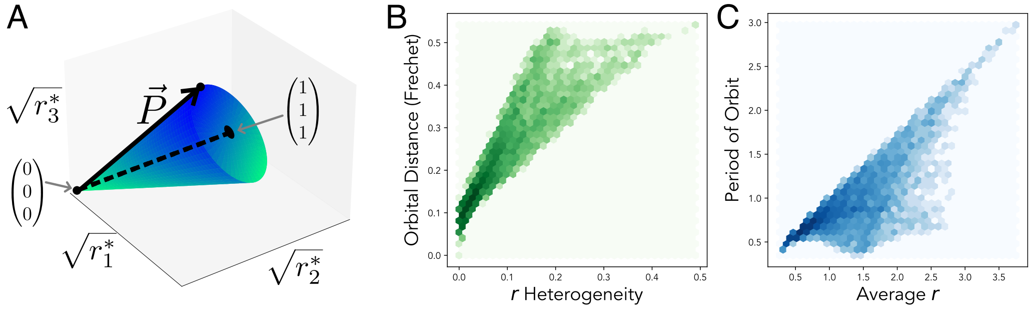

Fig. S2. A) A cone in the r_i space is used to define r heterogeneity. The angle θ measures the deviation of a point $\vec{P}$ from the homogenous direction (1, 1, 1) (dashed line). **B)** r heterogeneity vs the Fréchet distance for systems in the parameter search in Fig. 4 and Fig. 5. C) Average r vs the period of orbit for systems in the parameter search in Fig. 4 and Fig. 5.

Development • Supplementary information
