# Supplementary material for: A mathematical framework for measuring and tuning tempo in developmental gene regulatory networks
Source: Development. 2024 Jun 24;151(12):dev202950. doi: 10.1242/dev.202950 (PMC11234385; doi:10.1242/dev.202950)
Supplement: Supplementary file 1 [file develop-151-202950_review_history.pdf]

A mathematical framework for measuring and tuning tempo in developmental gene regulatory networks

Charlotte L Manser and Ruben Perez-Carrasco
DOI: 10.1242/dev.202950

Editor: Paul Francois

Review timeline
Original submission: 18 December 2023
Editorial decision: 20 February 2024
Resubmission: 12 April 2024
Accept: 13 May 2024

Original submission

First decision letter

MS ID#: DEVELOP/2023/202634

MS TITLE: A Mathematical Framework for Measuring and Tuning Tempo in Developmental Gene Regulatory Networks

AUTHORS: Charlotte Lily Manser and Ruben Perez-Carrasco

Dear Dr. Perez-Carrasco,

I have now received all the referees reports on the above manuscript, and have reached a decision. The referees' comments are appended below, or you can access them online: please go to BenchPress and click on the 'Manuscripts with Decisions' queue in the Author Area.

As you will see from their reports, the referees recognise the potential of your work, but they also raise significant concerns about it. Given the nature of these concerns, I am afraid I have little choice other than to reject the paper at this stage.

However, having evaluated the paper, I do recognise the potential importance of this work. I would therefore be prepared to consider as a new submission an extension of this study that contains new discussions and that address fully the major concerns of the referees. In particular, reviewers 2 and 3 were not fully convinced by the examples studied (e.g. represillator). Given the topic of our journal, I would advise better connecting the proposed framework to developmental biology. So the work required goes beyond a standard revision of the paper. Please bear in mind that the referees (who may be different from the present reviewers) will assess the novelty of your work in the context of all previous publications, including those published between now and the time of resubmission.

Reviewer 1

Advance Summary and Potential Significance to Field

This manuscript presents a mathematical framework for applying the concepts of orbital equivalence and orbital distance to the dynamics of developmental gene regulatory networks. This

© 2024. Published by The Company of Biologists under the terms of the Creative Commons Attribution License (https://creativecommons.org/licenses/by/4.0/).

is a valuable conceptual-mathematical contribution that will be useful for analysing both experimental data and mathematical models.

The framework is then applied to a simple repressilator model, demonstrating how it can be used to analyse the effects of model parameters both empirically and analytically. The interesting question of how best to compare different orbits / prefactors is left open for future work.

I have taken issue with the definitions/interpretations of some key terms (e.g. “tempo” and “function” of a GRN) and some of the reasoning used in the paper, as detailed in my major comments below. These problems are relatively superficial and can be addressed with changes to the text. If possible, I have also suggested the authors expand their framework by providing an additional comparative metric, quantifying how similar dynamical trajectories are in shape.

Finally, I should note my limitations as a reviewer for this interdisciplinary paper. I have focused on assessing the framework as applied to developmental biology rather than commenting on the accuracy or novelty of the maths. I trust that these aspects will be reviewed by someone with complementary expertise.

- Erik Clark

Comments for the author

Comments relating to terminology and definitions:
1. Defining “tempo” (conceptually).
The title of the manuscript is about “measuring and tuning tempo”, however the definition of tempo used seems significantly different from the concept as currently used in developmental biology. In music, tempo is simply beats per minute (i.e. changing the tempo preserves not just the sequence of notes, but also the relative lengths of the gaps between the notes). This accords with e.g. Rayon and Briscoe (2021) [Cross-species comparisons and in vitro models to study tempo in development and homeostasis], who describe developmental “allochrony” (their term for tempo) as follows: “In contrast with the broad meaning of heterochrony, which includes the disproportionate change in timescales of the same process … allochrony refers to proportionally scaled changes in the pace of development across species”.
In contrast, the authors write: “under the lens of dynamical systems, it becomes apparent that tempo changes are not limited to global constant organism-specific tempos, but more generally to changes in the local speed … at which the same gene dynamical landscape is traversed” (p3). (The music analogy would be preserving the same sequence of notes, but allowing all possible changes to the relative gaps in between them.) In my view, redefining “tempo” this way muddies the waters, as tempo is currently being used to refer to a specific phenomenon that maps nicely onto the musical definition.
I think it is fine (and indeed valuable!) to generalise the concept of dynamical speed changes as the authors do in establishing their mathematical-conceptual framework, but I think it would probably be preferable to reserve the specific term “tempo” for the special case of proportional temporal scaling, and designate a different term for the broader phenomenon. (Indeed, the authors raise this question as a live one: “when we claim that the differentiation of two cells is the same except for their tempo, what do we mean by the same?”)
Specifically, I would suggest that the authors revise the text to reframe their framework as generalising the concept of orbit-preserving trajectory changes, with “tempo” (sensu stricto) changes as a special case within this broad possibility space. Alternatively, make very clear that they are using a new and more expansive definition of tempo and argue convincingly that this is appropriate.
2. Defining “tempo” (mathematically).
If the recommendation in point 1 is taken up, then the “tempo” metric as currently described (equation 3) should be recognised as something more general and renamed accordingly.
In addition to this, given the aim of establishing a comprehensive mathematical framework, I think it would be very useful if the authors were able to come up with an additional metric that explicitly measures the proportionality of two different trajectories with equivalent orbits. (E.g., a simple tempo change would score 0, and changes affecting the shape of the trajectory would score >0.) This could be used in combination with equation 3 to independently compare the average rate and

© 2024. Published by The Company of Biologists under the terms of the Creative Commons Attribution License (https://creativecommons.org/licenses/by/4.0/).

proportionality of different dynamical trajectories. (I.e., capture and quantitate two distinct types of heterochrony for trajectories with equivalent orbits.)
3. Defining “function” of a GRN.
Throughout, the authors couch their mathematical framework explicitly in functional terms. (E.g. the section headers “Orbital equivalence: preserving the function of gene regulatory programs” (p1) and “Orbital distance: comparing the function of gene regulatory programs” (p3).) I think it’s important to be very careful here.
First, the “function” of a GRN can be used to describe very different things. There is the question of what kind of dynamical behaviour is produced by the GRN, and then there is the question of what this actually regulates in the developing organism. (“Activity-function” vs “use-function” in the terms of reference [32].) The manuscript under review is obviously concerned with the former question only and it would be helpful to clarify this. Also, given that “use-function” is not being addressed here, the use of the (undefined) term “phenotypic function” (p2) is ambiguous and probably better avoided.
Second, the basis for the particular mathematical definition of function used in the paper is given as follows: “Within the context of cell differentiation, the function of a genetic program is translated into the conserved sequence of relative expression of different genes, independently of the speed at which this sequence is travelled” (p1). This statement isn’t referenced or specifically justified, and I don’t think I would agree with it! As a consequence, it’s not clear to me that orbital equivalence really does “preserve the function of gene regulatory programs” or that “orbital distance” really does “compare the function of gene regulatory programs”. (To be clear, I think that the concepts of orbital equivalence and orbital distance, as applied to GRNs, are valuable, I just take issue with the functional terminology / interpretations.)
For example, take Figure 1A and 1B. In comment #1 I have already argued that the rate change in 1A (tempo change sensu stricto) is conceptually different from the non-proportional changes in 1B. However, whereas the authors describe both examples as “preserving the phenotypic function of the system”, in my view neither example preserves function, as in both cases the output patterns (“Phenotypes”) are significantly perturbed.
In contrast to the authors, my strong opinion is that in developing embryos, rates, not just orbits, are important for function. (This contributes to the evolutionary importance of tempo changes: if GRNs didn’t proportionally adapt their rates to changes in the duration of other developmental processes, you wouldn’t end up with a viable organism. Counter-intuitively, it’s not so much that the function of a GRN is rate-independent, but more that the “phenotypic” output depends crucially on relative rates [between a clock and a wavefront, say], which are themselves preserved via changes in tempo.)
Following from this, it’s also not clear to me that orbital distance is necessarily the best proxy for functional change in a GRN. Would preserving an orbit while severely distorting rates really preserve “function” better than a small change to the orbit which generally preserves the rates? (Say, compare the two patterns in Fig 1A with those of species A and B in Fig 1C.)
Specifically, I suggest the authors rethink carefully their use of functional terminology wherever it appears in the text. In my view it is justified to equate dynamical output (broadly defined) with [activity-]function, but not to assert that the sequence of states traversed is all that matters. In my view a significant benefit of the kind of mathematical framework offered by the authors is that it allows us to distinguish, measure and compare various different aspects of this dynamical output, and thereby investigate the contribution of each of them to development and evolution.
4. Defining “same landscape”.
At several places in the manuscript, there are sentences such as “the prefactor $\mu(\vec{x}, t)$ encodes all the possible changes that can be performed to the dynamical system that preserve intact the landscape.” (p3) and “Here we show how there are infinite available transformations of the dynamical system that preserve the same identical landscape.” (p10)
It would be helpful for the authors to very clearly define what they mean by “same landscape”. Again, like with point #1, this is a question of definitions rather than dynamical systems per se. For example, multiplying by a simple global (positive) prefactor preserves the direction and relative magnitude of the flow. Multiplying by a locally changing (positive) prefactor preserves the direction but not the relative magnitude of the flow. It’s not obvious to me that there is a single possible definition of “same landscape” - instead it seems like there could be multiple, more or less expansive, definitions of “same landscape”, depending on which specific features are preserved.
Other major comments:
5. Choice of distance metric.

© 2024. Published by The Company of Biologists under the terms of the Creative Commons Attribution License (https://creativecommons.org/licenses/by/4.0/).

As the authors write (p3): “we will evaluate the distance of two orbits using the Fréchet distance…Several alternative definitions of orbital distance are possible…Ultimately, the choice of orbital distance needs to address how similar the function of two systems is.”
This is an important and interesting question for future investigation. As such, I think that the SI section “Alternative definitions of orbital distance” plus Figure S1 should actually be moved to the main paper. (From a biological perspective, the DTW metric seems the most intuitive of the three to me.)
"despite these differences, we observe similar results when using Fréchet, Hausdorff and DTW to compare against analytical measures of orbital distance through the prefactor" (SI p1)
This conclusion is not clear to me from the results shown. In addition to the different rank orders mentioned in the text, the relationship between prefactor heterogeneity and orbital distance is roughly linear for Fréchet but not Hausdorff or DTW. What is meant by “similar” here?
6. Analytical orbital distance.
“we can also attempt to define an analytical orbital distance using the definition of orbital equivalence….an orbital distance can be defined by quantifying the heterogeneity of the components of the prefactor vector… To compare with the results from the Fréchet distance, we can define an orbital prefactor heterogeneity distance as the maximum heterogeneity along the orbit” (p5)
The choice of metrics raises similar issues to comment 5 above, and could be further discussed.
“Using our analytical definition of prefactor heterogeneity Dσ… and comparing it with the orbital distance calculated by numerical integrations of the perturbed systems, we observed a linear relationship between the two (Fig. 5A). This relationship reveals an analytical means to predict changes in the orbit’s shape” (p8)
This is a nice result, though it’s unclear to me how much it will generalise, especially given the dependence on choice of distance metric, as discussed in comment #5, even for the simple repressilator example. This could be discussed. (Also, perhaps this is too pedantic, but the phrasing above is a little ambiguous - the authors have provided an analytic means to predict the magnitude of changes in the orbit’s shape, but not what those changes are per se.)
“we investigated whether the period of oscillations in the perturbed system could be predicted by analyzing only the prefactor of the new system. In this analysis, we found that the average prefactor serves as a reliable predictor of oscillation periods (Fig. 5B).” (p8) “In particular, we identified that the average of the composed dimerization rates completely predicts the average prefactors, and consequently the period of the resulting orbit (Fig. 5E and Fig. S.2B).” (p9)
It is again a nice result to find predictive relationships between particular (combinations of) parameter values and the average prefactor and, in turn, oscillation period. But the language used (especially “completely predicts”) seems too strong, given the y axis variation in figs 5B, 5E, and S2C.
“While there are different choices for this comparison [between the perturbed and unperturbed rates], for the case of the repressilator, we found that the best correlation against prefactor heterogeneity was achieved through the angle θ between the homogeneous case (1, 1, 1) and the vector $(\sqrt{r1},\sqrt{r2},\sqrt{r3})$.” (SI p4)
While I have no problem with the choice itself, given that the nature of the relationship between r heterogeneity and prefactor heterogeneity is described in the main text as a result, it may be worth noting that the choice of metric for r heterogeneity was explicitly influenced by which one gave the best correlation with prefactor heterogeneity.
7. Overall manuscript structure.
The figures are attractive and communicate the main concepts and results well. However, to improve the impact of the paper, I think the text could be made more inviting to a developmental biology audience. (I.e., a bit more explanation of the mathematical concepts/terminology and more text helping to lead the reader through the mathematical results.) Possibly some of the repressilator equations and their rearrangements could be moved to the SI and replaced with a more-text based explanation of the key analytical results and their importance; I don’t think it’s really necessary to follow the maths in detail in order to appreciate the utility of applying the framework (and some of the potential audience may not have the facility or inclination to follow the details anyway). I’m also not sure the current structure of the paper (Materials and Methods vs Results) is the most appropriate, when really what we have is the justification for a general mathematical-conceptual framework followed by its application to a specific case.
Minor comments

© 2024. Published by The Company of Biologists under the terms of the Creative Commons Attribution License (https://creativecommons.org/licenses/by/4.0/).

1. “This identification of gene expression orbits with their core functional activity harmonizes with the proposed concept of dynamical modularity proposed by Jaeger and Monk [32].” (p3). It would be helpful to the reader to quote the definition being referenced. (And see Major comment #3).
2. “For the purpose of this study, we will describe gene expression as a set of biochemical reactions that can be modelled as a system of ordinary differential equations” (p3) ... “One immediate result from the orbital equivalence framework is that identical global rescaling of protein production and degradation rates of the transcription factors of a given gene regulatory network will preserve the orbit while controlling tempo…This result applies to any given regulatory network independently of the complexity of its topology (see Fig. 3), suggesting a robust mechanism to tune tempo of a regulatory system, allowing for evolutionary strategies.” (p5)
The result described in the p5 quote applies to ODE models of GRNs as described in p3. It is perhaps worth pointing out that the result will not necessarily generalise to other kinds of models (or real biological systems). For example, I can imagine that the magnitude of protein production and degradation rates relative to say splicing delays or transcriptional noise might affect the orbit.
3. The following sentences could be made clearer:
“This strategy offers a mechanistic insight into gene expression dynamics by establishing the rules governing reaction rates among biochemical species.” (p1) “usually fixing the location and change of steady states and separatrices of the landscape” (p10)
4. There seems to be inconsistency in the way that ri is used in the following pair of sentences (first as individual rates r1, r2, r3, and then as the “ensemble” of the three rates together):
“Initial inspection of the role that individual rates have on the prefactor reveals that, while there is some correlation between ri and the resulting prefactor heterogeneity and average prefactor, there is not a strong predictive relationship (Fig. 5C, D). These results suggest that the effect that changes of prefactor have on the orbit originate from a combination of perturbations in the ensemble of rates included in ri.” (p8)
5. Some typos in text:
“Matertials and Methods” (p1)
“Along this manuscript” (p3)
“Schematic showing how a dynamical system f($\vec{}$x, t)...same orbits g($\vec{}$x, t)” (p4) [f and g should be vectors?]
“Despite not corresponding with [a] particular developmental biology example” (p5)
“each rate rref i was perturbed with a uniform fold change returning new rates ri = rref” (p7) [is there a coefficient missing?]
“Since the three genes have different prefactors μi(pi), that [which] depend on the parameters and gene expression” (p8)
6. Typo in equation S.2: Tf should be Tg?
7. Figure 3: the rightmost plot is ambiguous; it’s not clear that the four different lines are all meant to represent the same orbit.
8. It took me a second look to notice that there were both kappas and ks being used in section 3.2 (p10); perhaps a more distinct set of symbols could be chosen?

Reviewer 2

Advance Summary and Potential Significance to Field

The manuscript presents a different conceptual framework by investigating the phase-space trajectories and
the speed with which they evolve in phase space in contrast to the classical attractor framework the
emphasizes only the end state. In my opinion, this framework would be generally also very attractive for the
study and interpretation of experimental data, especially for, but not only limited to developmental questions.

Comments for the author

The manuscript by Manser and Perez-Carrasco introduces a mathematical framework for measuring and tuning tempo in GRN models, with the aim to address how timing of cell decisions is precisely controlled and which factors play a role. The manuscript presents a different conceptual

© 2024. Published by The Company of Biologists under the terms of the Creative Commons Attribution License (https://creativecommons.org/licenses/by/4.0/).

framework by investigating the phase-space trajectories and the speed with which they evolve in phase space in contrast to the classical attractor framework the emphasizes only the end state. In my opinion, this framework would be generally also very attractive for the study and interpretation of experimental data, especially but not only limited to developmental questions, and therefore I strongly support the publication of this work. However, I would like to ask the authors to consider some conceptual clarifications/changes before.
The authors write that the method focuses on transients, however both examples which they give – repressilator and the toggle switch correspond to attractor-based models, thus the transients only refer to asymptotic transients that quickly converge to the attractor. I find this generally contradictory to the author's statements in the introduction. The examples they cited in terms of transients i.e. Ref.30 and 41 however refer to 'long' transients - the system dynamic's is maintained away from the steady state for prolonged intervals of time and thereby characterized by non-asymptotic dynamics (following definition by Hastings et al., Science 361, 2018; Nandan et al., PloS Comp. Biol. 1011388, 2023). What has been the motivation to represent the current models – asymptotic transients and no clear/explicit relation to developmental processes? I strongly suggest to the authors to consider (at least) one example that features non-asymptotic transients. It would be as well advisable to consider models that can be directly related to developmental processes – for example, work from Francois et al. (eLife 55778, 2020) has a relevant oscillatory dynamics models where one could analyze both – asymptotic an non-asymptotic dynamics. Would be of great interest in my opinion to have equivalent comparison for non-oscillatory models – consider examples from ref.41 or Manu et al., PlS Comp. Biol 5(3): e1000303 – for non-asymptotic transient dynamics.
In terms of the way the manuscript is presented, the Frechet distance is not defined neither in the main nor in the supplementary material, and conceptually is already used in Fig.1 - please define it correspondingly, and please shortly describe the conceptual difference of this measure with respect to the others mentioned / shown in supplementary material.
Figure 2 – I am really unclear why toggle switch model is being used to convey the message. This can be done also schematically. Fig. 2 has been also referenced in the text before Fig1C – as my understanding is that both Figs.1,2 represent clarification of the concept, I suggest to the authors to optimize the description and presentation and show it in a more concise format, and not to introduce a model to convey clarification of the concept. The same problem I honestly encountered with Fig. 3 – it carries a very important message, that global rescaling of protein production and degradation rates of the transcription factors of a given gene regulatory network will preserve the orbit while controlling tempo, but it is unclear whether this is a scheme or these are actual results. This comes from their analytical approach shown in Supplementary – then it should be clearly and more extensively described, and maybe the authors can show to numerical examples (maybe from the models they use later on in the paper).
In terms of presentation of the result – I personally find the manuscript confusing to read, whereas I think the results themselves are very strong. For example, the description of Fig. 4 -n I would suggest to invest in relating E to F-G, add the relevant periods and distances on the axes and discuss in greater extent the implication of these findings. Same goes for Fig. 5.

Reviewer 3

Advance Summary and Potential Significance to Field

This theoretical study is concerned with the control of tempo in gene regulatory networks. As the authors note, several recent experimental studies have asked how homologous processes can take place at a different pace in different species, and a theoretical framework to approach this question should be of interest. The authors propose that such a framework may be found in the notion of orbital equivalence:
factoring out the pace of system evolution, two dynamical systems can be considered equivalent if they possess the same orbits; otherwise, it is proposed that a measure of their non-equivalence can be constructed from a measure of distances between orbits.

Comments for the author

Although the question laid out by the authors is of interest, my evaluation is that the insights delivered by their manuscript remain limited. On the one hand, it is my impression that the

© 2024. Published by The Company of Biologists under the terms of the Creative Commons Attribution License (https://creativecommons.org/licenses/by/4.0/).

definitions of system equivalence and distance measures considered are well defined only for systems with a single, periodic orbit. How does one define a distance between systems with multiple attractors, like the bistable system in Fig. 3? How should different trajectories, originating from different initial conditions, be weighted? Also, in defining distances, should one allow for a rescaling of gene expression levels, to allow that these could in principle be altered without changing the overall dynamics of a system? What about more elaborate mappings in gene expression space that preserve function? The discussion mentions "comparing orbits directly from gene expression data", but no specific discussion of applications to actual experimental data is given.
I also did not find the examples intended to demonstrate the framework very compelling. The first of what is presented in the abstract as two case studies is just a brief statement of a default model for changes in tempo - the global modulation of all reaction rates (Section 3.1). Figure 3 illustrates this with a time-dependent modulation of reaction rates, but would a state-dependent modulation of the rates (mu(x) instead of mu(t)) not be equally relevant, where systems with periodic orbits are concerned? The second example is mostly taken from a published study of a particular gene network, and does not appear to me to deliver insights that are particularly new, general, or convincing. To take one example, in their discussion of this system, the authors point to certain combinations of parameters (r_i) as levers to control tempo (Section 3.2); but, if I am not mistaken, the prefactors mu_i from eq. 11, which should vary coherently to preserve orbits, also depend on the sytem state (on the p_i), thus I am not clear that is choice is so well justified, or how this is supposed to give an analytical bearing on the problem (cf. first paragraph of Section 3.3).

Author response to reviewers' comments

A Mathematical Framework for Measuring and Tuning Tempo in Developmental Gene Regulatory Networks

Charlotte Manser, Ruben Perez-Carrasco

April 2024

We thank the referees for the overall positive response. We have improved the paper following the comments of the referees. Some major changes include the improvement of the connection between tempo and function, as well as a new section where we apply the framework to a specific developmental biology scenario. In particular we studied tempo tuning in the case of neural precursor differentiation, one of the main systems in which tempo has been interrogated experimentally and computationally [13]. We hope that this inclusion helps to cement this manuscript’s place in Development, especially given the positive reception from the reviewers.

1 Reviewer 1

> This manuscript presents a mathematical framework for applying the concepts of orbital equivalence and orbital distance to the dynamics of developmental gene regulatory networks. This is a valuable conceptual-mathematical contribution that will be useful for analysing both experimental data and mathematical models.

We thank the reviewer for their positive comments. In addition, we sincerely appreciate the referee’s meticulous review and insightful comments, which have significantly contributed to the refinement of our manuscript.

Comments relating to terminology and definitions:

1.1 Definition of “tempo”

The title of the manuscript is about “measuring and tuning tempo”, however the defini- tion of

© 2024. Published by The Company of Biologists under the terms of the Creative Commons Attribution License (https://creativecommons.org/licenses/by/4.0/).

tempo used seems significantly different from the concept currently used in devel- opmental biology. In music, tempo is simply beats per minute (i.e. changing the tempo preserves not just the sequence of notes, but also the relative lengths of the gaps between the notes). This accords with e.g. Rayon and Briscoe (2021) [Cross-species comparisons and in vitro models to study tempo in development and homeostasis], who describe de-velopmental "allochrony" (their term for tempo) as follows: "In contrast with the broad meaning of heterochrony, which includes the disproportionate change in timescales of the same process . . . allochrony refers to proportionally scaled changes in the pace of development across species".

In contrast, the authors write: "under the lens of dynamical systems, it becomes apparent that tempo changes are not limited to global constant organism-specific tempos, but more generally to changes in the local speed . . . at which the same gene dynamical landscape is traversed" (p3). (The music analogy would be preserving the same sequence of notes, but allowing all possible changes to the relative gaps in between them.) In my view, redefining "tempo" this way muddies the waters, as tempo is currently being used to refer to a specific phenomenon that maps nicely onto the musical definition.

I think it is fine (and indeed valuable!) to generalise the concept of dynamical speed changes as the authors do in establishing their mathematical-conceptual framework, but I think it would probably be preferable to reserve the specific term "tempo" for the special case of proportional temporal scaling, and designate a different term for the broader phenomenon. (Indeed, the authors raise this question as a live one: "when we claim that the differentiation of two cells is the same except for their tempo, what do we mean by the same?")

Specifically, I would suggest that the authors revise the text to reframe their framework as generalising the concept of orbit-preserving trajectory changes, with "tempo" (sensu stricto) changes as a special case within this broad possibility space. Alternatively, make very clear that they are using a new and more expansive definition of tempo and argue convincingly that this is appropriate.

If the recommendation in point 1 is taken up, then the "tempo" metric as currently described (equation 3) should be recognised as something more general and renamed accordingly.

We agree with the author that our use of 'tempo' is a more general than the concept of 'allochrony' which the author identifies as the current definition of tempo in developmental biology. However, we suggest that allochrony is not the only (and likely not the default) mode of tempo change. This harmonizes with the definition given by Rayon and Briscoe (2021), who define tempo as "the speed of these processes", and do not identify 'tempo' with 'allochrony'. Similarly, other reviews such as [17], Diaz-Cuandros and Pourqui´e dis- tinguish both allochrony and heterochrony, as the mode of non-uniform differences between developmental events, but without associating 'tempo' to any of both terms. In addition, in the seminal work where the word tempo is introduced by Rayon et al (2021), there is the observation that there are minor differences in the temporal progression of different genes along the cascade, compatible with the idea that even allochrony could include small varia- tions in speed along the same sequence of gene expression. In addition we believe that our definition of tempo is fully compatible with the musical analogy, where in the same musical piece is very common to find changes in tempo (discrete and continuously) across the piece. We have connected the ideas of tempo, heterochrony and alochrony in the context of our manuscript after the calculation of the average prefactor (Eq.3), including also a specific comment on the musical analogy.

In addition to this, given the aim of establishing a comprehensive mathematical frame- work, I think it would be very useful if the authors were able to come up with an ad- ditional metric that explicitly measures the proportionality of two different trajectories with equivalent orbits. (E.g., a simple tempo change would score 0, and changes affecting the shape of the trajectory would score >0.) This could be used in combination with equation 3 to independently compare the average rate and proportionality of different dynamical trajectories. (I.e., capture and quantitate two distinct types of heterochrony for trajectories with equivalent orbits.)

We thank the reviewer for this idea, we have included such a metric after eq.(3). The metric

© 2024. Published by The Company of Biologists under the terms of the Creative Commons Attribution License (https://creativecommons.org/licenses/by/4.0/).

measures the deviation of the prefactor μ from its average along an orbit $\bar{\mu}$, and can be used to measure how far from exact alochrony is the tempo change.

1.2 Defining "function" of a GRN

Throughout, the authors couch their mathematical framework explicitly in functional terms. (E.g. the section headers "Orbital equivalence: preserving the function of gene regulatory programs" (p1) and "Orbital distance: comparing the function of gene regu- latory programs" (p3).) I think it's important to be very careful here.
First, the "function" of a GRN can be used to describe very different things. There is the question of what kind of dynamical behaviour is produced by the GRN, and then there is the question of what this actually regulates in the developing organism. ("Activity- function" vs "use-function" in the terms of reference [32].) The manuscript under review is obviously concerned with the former question only and it would be helpful to clarify this.

We agree with the reviewer that 'biological function' is a philosophically loaded term that needs careful treatment. We have added a paragraph on page 3 to make it clear that we are concerned only with activity-function, and that other useful definitions exist. We have also included arguments as to why we chose activity-function.

Also, given that "use-function" is not being addressed here, the use of the (undefined) term "phenotypic function" (p2) is ambiguous and probably better avoided.

We agree. We have removed references to "phenotypic function" along the text.

Second, the basis for the particular mathematical definition of function used in the paper is given as follows: "Within the context of cell differentiation, the function of a genetic program is translated into the conserved sequence of relative expression of different genes, independently of the speed at which this sequence is travelled" (p1). This statement isn't referenced or specifically justified, and I don't think I would agree with it! As a consequence, it's not clear to me that orbital equivalence really does "preserve the function of gene regulatory programs" or that "orbital distance" really does "compare the function of gene regulatory programs". (To be clear, I think that the concepts of orbital equivalence and orbital distance, as applied to GRNs, are valuable, I just take issue with the functional terminology / interpretations.)

We agree that the explored mathematical definition of function used in the paper is not unique. We have made it clearer in the text that this is a choice. We have also added a paragraph which gives the motivation for our choice in section 2.1.

For example, take Figure 1A and 1B. In comment (1) I have already argued that the rate change in 1A (tempo change sensu stricto) is conceptually different from the non- proportional changes in 1B. However, whereas the authors describe both examples as "preserving the phenotypic function of the system", in my view neither example preserves function, as in both cases the output patterns ("Phenotypes") are significantly perturbed.

We agree with the author that the phenotypes of the figure are different from each other. For this reason we have reinforced in the figure legend how these outputs demonstrate conserved functions based on our definition.

In contrast to the authors, my strong opinion is that in developing embryos, rates, not just orbits, are important for function. (This contributes to the evolutionary importance of tempo changes: if GRNs didn't proportionally adapt their rates to changes in the duration of other developmental processes, you wouldn't end up with a viable organism. Counter-intuitively, it's not so much that the function of a GRN is rate-independent, but more that the "phenotypic" output depends crucially on relative rates [between a clock and a wavefront, say], which are themselves preserved via changes in tempo.)
Following from this, it's also not clear to me that orbital distance is necessarily the best proxy for functional change in a GRN. Would preserving an orbit while severely distorting rates really preserve "function" better than a small change to the orbit which generally preserves the

© 2024. Published by The Company of Biologists under the terms of the Creative Commons Attribution License (https://creativecommons.org/licenses/by/4.0/).

rates? (Say, compare the two patterns in Fig 1A with those of species A and B in Fig 1C.)

We agree with the reviewer that the rate at which an orbit is trasversed are very important for function in developing embryos. We believe that the reviewer's comments are more relevant to use-function, and therefore to a broader question of how interconnecting processes affect one another in a whole embryo. Since we are looking specifically at the activity-function of dynamical modules, we are not assessing the wider impact that the change in the dynamic function will have in the developing embryo. In addition, since it has been demonstrated that the current favoured processes to be studied for species-specific tempo (somitogenesis and motor neuron differentiation) are both cell-intrinsic, we believe that this modularity assumption is justified. Overall, our methodology allows us to separate these two different aspects, rates (tempo) and geometrical orbit shape, the perturbation of which can be used to study in the future their different phenotypic implications in the embryo. We have added this observation to the discussion of the manuscript.

Specifically, I suggest the authors rethink carefully their use of functional terminology wherever it appears in the text. In my view it is justified to equate dynamical output (broadly defined) with [activity-]function, but not to assert that the sequence of states traversed is all that matters. In my view a significant benefit of the kind of mathemat- ical framework offered by the authors is that it allows us to distinguish, measure and compare various different aspects of this dynamical output, and thereby investigate the contribution of each of them to development and evolution.

We have further elaborated on why we have chosen this definition (namely that it is a mathematical translation of Jaeger's dynamical modules) in the new paragraph in section 2.1 concerning function, and clarifying some of the references to "function" along the text.

1.3 Defining "same landscape"

At several places in the manuscript, there are sentences such as "the prefactor $\vec{\mu}(x, t)$ encodes all the possible changes that can be performed to the dynamical system that preserve intact the landscape." (p3) and "Here we show how there are infinite available transformations of the dynamical system that preserve the same identical landscape." (p10)

It would be helpful for the authors to very clearly define what they mean by "same land- scape". Again, like with point (1), this is a question of definitions rather than dynamical systems per se. For example, multiplying by a simple global (positive) prefactor preserves the direction and relative magnitude of the flow. Multiplying by a locally changing (pos- itive) prefactor preserves the direction but not the relative magnitude of the flow. It's not obvious to me that there is a single possible definition of "same landscape" - in- stead it seems like there could be multiple, more or less expansive, definitions of "same landscape", depending on which specific features are preserved.

The word landscape is useful to convey the idea of temporal evolution of a dynamical system in a biological context, but it is flawed from a mathematical point of view where a strict definition of landscape requires a conservative (curl-free) dynamical system. In our case we refer to landscape in a Waddingtonian sense, in which number of available states, the position and span of valleys of attraction, and the sequence of cell states are preserved. We have clarified this in the text following the suggestion of the referee, making explicit reference to the "direction of the flow".

Other major comments:

1.4 Choice of distance metric

As the authors write (p3): "we will evaluate the distance of two orbits using the Fr´echet distance. . . Several alternative definitions of orbital distance are possible. . . Ultimately, the choice of orbital distance needs to address how similar the function of two systems is."
This is an important and interesting question for future investigation. As such, I think that the SI section "Alternative definitions of orbital distance" plus Figure S1 should actually be moved to the main paper. (From a biological perspective, the DTW metric seems the most intuitive of the three to me.)

We agree with the reviewer that this is an interesting aspect of our research, and especially that

© 2024. Published by The Company of Biologists under the terms of the Creative Commons Attribution License (https://creativecommons.org/licenses/by/4.0/).

DTW should be addressed in the main text because of its popularity in the develop- mental biology community. A paragraph mentioning DTW and its differences from Fr´echet, and ultimately why we chose Fr´echet distance, has been added to the appropriate section (2.2). The reason we chose Fr´echet over DTW is that the former is better at penalizing local dramatic deformations of the orbit as opposed to a small orbit shift. We have not moved the entire section from the SI to the main text out of concern for the paper getting too long and overly mathematical, but we hope our added content brings enough insight.

> ”despite these differences, we observe similar results when using Fr´echet, Hausdorff and DTW to compare against analytical measures of orbital distance through the prefactor” (SI p1)
> This conclusion is not clear to me from the results shown. In addition to the different rank orders mentioned in the text, the relationship between prefactor heterogeneity and orbital distance is roughly linear for Fr´echet but not Hausdorff or DTW. What is meant by “similar” here?

By ‘similar’ we mean that all distances correlate positively, this has been clarified in the supplementary text.

1.5 Analytical orbital distance

> “we can also attempt to define an analytical orbital distance using the definition of orbital equivalence. an orbital distance can be defined by quantifying the heterogeneity of the components of the prefactor vector. . . To compare with the results from the Fr´echet distance, we can define an orbital prefactor heterogeneity distance as the maximum heterogeneity along the orbit” (p5)
> The choice of metrics raises similar issues to comment 5 above, and could be further discussed.

This comment should be clear after the additional discussion on the Fr´echet distance included in section 2.2.

> “Using our analytical definition of prefactor heterogeneity $D\sigma$. . . and comparing it with the orbital distance calculated by numerical integrations of the perturbed systems, we observed a linear relationship between the two (Fig. 5A). This relationship reveals an analytical means to predict changes in the orbit’s shape” (p8)
> This is a nice result, though it’s unclear to me how much it will generalise, especially given the dependence on choice of distance metric, as discussed in comment (5), even for the simple repressilator example. This could be discussed. (Also, perhaps this is too pedantic, but the phrasing above is a little ambiguous - the authors have provided an analytic means to predict the magnitude of changes in the orbit’s shape, but not what those changes are per se.)

The positive correlation of Fr´echet distance with the prefactor heterogeneity definition (Eq. 5) is expected to generalize given that they are designed to capture the same features that orbital equivalence. We agree that the linear relationship is not straightforward, and we expect that it this linearity will be broken for different networks. We believe that exploring this further would require more substantial research out of the scope of this paper. Regarding the phrasing of the sentence, it has been ammended to to make it more precise.

> “we investigated whether the period of oscillations in the perturbed system could be predicted by analyzing only the prefactor of the new system. In this analysis, we found that the average prefactor serves as a reliable predictor of oscillation periods (Fig. 5B).” (p8) “In particular, we identified that the average of the composed dimerization rates completely predicts the average prefactors, and consequently the period of the resulting orbit (Fig. 5E and Fig. S.2B).” (p9)
> It is again a nice result to find predictive relationships between particular (combinations of) parameter values and the average prefactor and, in turn, oscillation period. But the language used (especially “completely predicts”) seems too strong, given the y axis variation in figs 5B, 5E, and S2C.

The strength of these statements has been moderated to reflect a more cautious scientific claim.

© 2024. Published by The Company of Biologists under the terms of the Creative Commons Attribution License (https://creativecommons.org/licenses/by/4.0/).

"While there are different choices for this comparison [between the perturbed and unperturbed rates], for the case of the repressilator, we found that the best correlation against prefactor heterogeneity was achieved through the angle σ between the homogeneous case (1, 1, 1) and the vector $(sqrtr_1, sqrtr_2, sqrtr_3)$." (SI p4)
While I have no problem with the choice itself, given that the nature of the relationship between r heterogeneity and prefactor heterogeneity is described in the main text as aresult, it may be worth noting that the choice of metric for r heterogeneity was explicitly influenced by which one gave the best correlation with prefactor heterogeneity.

We have made it more clear in the main text that r heterogeneity was chosen because of its correlation with prefactor heterogeneity.

1.6 Overall manuscript structure

The figures are attractive and communicate the main concepts and results well. However, to improve the impact of the paper, I think the text could be made more inviting to a developmental biology audience. (I.e., a bit more explanation of the mathematical concepts/terminology and more text helping to lead the reader through the mathematical results.) Possibly some of the repressilator equations and their rearrangements could be moved to the SI and replaced with a more-text based explanation of the key analytical results and their importance; I don't think it's really necessary to follow the maths in detail in order to appreciate the utility of applying the framework (and some of the potential audience may not have the facility or inclination to follow the details anyway).

To better engage our readers from the developmental biology community, we have introduced a new section that demonstrates our methodology's application to the neural progen- itor differentiation gene regulatory network. This section emphasizes the impact of protein production on the existing proposed models that control developmental tempo.

We recognize the referee's concern about ensuring the paper's accessibility to a biological audience. We were very careful in the first version of the manuscript to include only the minimal equations required, keeping the main text focused on the biology. After discussion among the authors, we still believe that the included equations in the main text are the minimal subset of equations. First, our discussion on the exploration of parameter space, specifically the dimerization fluxes ri, would be incomplete and confusing without a detailed motivation from the mathematical derivation. Second, the especific derivation of the quasi- steady-state conditions is essential for the mathematical framework we propose, justifying its presence in the main text. While we have kept the equations in the text, we have further clarified several concepts as proposed by the referee. Our goal is to make the text not only more accessible to non-specialists but also to maintain its relevance and utility for the readership of Development with a more mathematical background. We hope these adjustments have made the article more approachable for all readers.

I'm also not sure the current structure of the paper (Materials and Methods vs Re- sults) is the most appropriate, when really what we have is the justification for a general mathematical-conceptual framework followed by its application to a specific case.

We agree with the reviewer. We have kept the ordering and organisation of the text the same, but renamed the section 'Materials and Methods' to 'A Mathematical Framework for Function and Tempo' and 'Results' to 'Applications'.

1.7 Minor comments

1. "This identification of gene expression orbits with their core functional activity harmo- nizes with the proposed concept of dynamical modularity proposed by Jaeger and Monk [32]." (p3). It would be helpful to the reader to quote the definition being referenced. (And see Major comment (3)).

The quote has been inserted in the new version of the manuscript.

2. "For the purpose of this study, we will describe gene expression as a set of biochemical reactions that can be modelled as a system of ordinary differential equations" (p3) ... "One

© 2024. Published by The Company of Biologists under the terms of the Creative Commons Attribution License (https://creativecommons.org/licenses/by/4.0/).

immediate result from the orbital equivalence framework is that identical global rescaling of protein production and degradation rates of the transcription factors of a given gene regulatory network will preserve the orbit while controlling tempo. . . This result applies to any given regulatory network independently of the complexity of its topology (see Fig. 3), suggesting a robust mechanism to tune tempo of a regulatory system, allowing for evolutionary strategies.” (p5)
The result described in the p5 quote applies to ODE models of GRNs as described in p3. It is perhaps worth pointing out that the result will not necessarily generalise to other kinds of models (or real biological systems). For example, I can imagine that the magnitude of protein production and degradation rates relative to say splicing delays or transcriptional noise might affect the orbit.

The result holds for any gene regulatory network that can be described by an ODE where the rate-limiting step is protein production and degradation, we have made this clearer thorugh the new section of neural differentiation, and also in the discussion.

3. The following sentences could be made clearer:

“This strategy offers a mechanistic insight into gene expression dynamics by establishing the rules governing reaction rates among biochemical species.” (p1)

“usually fixing the location and change of steady states and separatrices of the landscape” (p10)

We have removed the first sentence since it did not contribute to the flow and message of the paragraph. In the second sentence we have replaced the word “separatrices” by “basins of attractions” to make it more readable.

4. There seems to be inconsistency in the way that ri is used in the following pair of sentences (first as individual rates r1, r2, r3, and then as the “ensemble” of the three rates together):
“Initial inspection of the role that individual rates have on the prefactor reveals that, while there is some correlation between ri and the resulting prefactor heterogeneity and average prefactor, there is not a strong predictive relationship (Fig. 5C, D). These results suggest that the effect that changes of prefactor have on the orbit originate from a combination of perturbations in the ensemble of rates included in ri.” (p8)

In the new version of the manuscript we have made explicit when we are talking of individual vs. ensemble r_i

1.8 Some typos in text

“Matertials and Methods” (p1) “Along this manuscript” (p3)
“Schematic showing how a dynamical system $f(\vec{x}, t)$...same orbits $g(\vec{x}, t)$” (p4) [f and g should be vectors?]
“Despite not corresponding with [a] particular developmental biology example” (p5)
“each rate rref i was perturbed with a uniform fold change returning new rates ri = rref” (p7) [is there a coefficient missing?]
“Since the three genes have different prefactors $\mu_i(\pi)$, that [which] depend on the param- eters and gene expression” (p8)
6. Typo in equation S.2: Tf should be Tg?
7. Figure 3: the rightmost plot is ambiguous; it’s not clear that the four different lines are all meant to represent the same orbit.
8. It took me a second look to notice that there were both kappas and ks being usedinsection 3.2 (p10); perhaps a more distinct set of symbols could be chosen?

We thank the reviewer for finding these typos, they have been corrected in the updated version of the manuscript.

2 Reviewer 2

The manuscript by Manser and Perez-Carrasco introduces a mathematical framework for

© 2024. Published by The Company of Biologists under the terms of the Creative Commons Attribution License (https://creativecommons.org/licenses/by/4.0/).

measuring and tuning tempo in GRN models, with the aim to address how timing of cell decisions is precisely controlled and which factors play a role. The manuscript presents a different conceptual framework by investigating the phase-space trajectories and the speed with which they evolve in phase space in contrast to the classical attractor framework the emphasizes only the end state. In my opinion, this framework would be generally also very attractive for the study and interpretation of experimental data, especially but not only limited to developmental questions, and therefore I strongly support the publication of this work. However, I would like to ask the authors to consider some conceptual clarifications/changes before.

We thank the reviewer for their positive comments on the paper and for their enthusiasm about publishing it.

The authors write that the method focuses on transients, however both examples which they give – repressilator and the toggle switch correspond to attractor-based models, thus the transients only refer to asymptotic transients that quickly converge to the attractor. I find this generally contradictory to the author's statements in the introduction. The examples they cited in terms of transients i.e. Ref.30 and 41 however refer to 'long' transients - the system dynamic's is maintained away from the steady state for prolonged intervals of time and thereby characterized by non-asymptotic dynamics (following defi- nition by Hastings et al., Science 361, 2018; Nandan et al., PloS Comp. Biol. 1011388, 2023). What has been the motivation to represent the current models – asymptotic tran- sients and no clear/explicit relation to developmental processes? I strongly suggest to the authors to consider (at least) one example that features non-asymptotic transients. It would be as well advisable to consider models that can be directly related to devel- opmental processes – for example, work from Francois et al. (eLife 55778, 2020) has a relevant oscillatory dynamics models where one could analyze both – asymptotic an non-asymptotic dynamics. Would be of great interest in my opinion to have equivalent comparison for non-oscillatory models – consider examples from ref.41 or Manu et al., PlS Comp. Biol 5(3): e1000303 – for non-asymptotic transient dynamics.

We agree that an example more relevant to development, with non-oscillatory transients, would convey our results better. Consequently we have rewritten our first application to the neural precursor differentiation gene regulatory network. We have used this network since it has recently been used to explain temporal differences in the development of neuronal differentiatiopn between human and mouse. In this particular case, the GRN is in charge of the sequential expression of three genes that eventually converge to a steady state.

In terms of the way the manuscript is presented, the Frechet distance is not defined neither in the main nor in the supplementary material, and conceptually is already used in Fig.1 - please define it correspondingly, and please shortly describe the conceptual difference of this measure with respect to the others mentioned/shown in supplementary material.

We agree that the Fr´echet distance should be defined, and thank the reviewer for noticing that we had overlooked it. We have added an intuitive definition in the main text (section 2.2) and a formal definition in the supplementary information. We have also shortly described its conceptual difference to the most well-known alternative, dynamical time warping, in the same section. To stop the main text from being too long, we've kept comparisons to other measures in the supplementary material.

Figure 2 – I am really unclear why toggle switch model is being used to convey the message. This can be done also schematically. Fig. 2 has been also referenced in the text before Fig1C – as my understanding is that both Figs.1,2 represent clarification of the concept, I suggest to the authors to optimize the description and presentation and show it in a more concise format, and not to introduce a model to convey clarification of the concept.

The intent of the first section of the manuscript is not just to explain qualitatively the concept but also to set its mathematical basis. We have renamed that section to "A Mathematical Framework for Function and Tempo" to make it more explicit. While Figure 1 is purely a schematic for the concept of orbital equivalence, this is not the case for Figure 2, where we wanted to show how the concept of prefactor heterogeneity works in a specific example. We have chosen the

© 2024. Published by The Company of Biologists under the terms of the Creative Commons Attribution License (https://creativecommons.org/licenses/by/4.0/).

toggle switch to show a paradigmatic example where two attracting steady states are available. We believe that having the network allows the reader to understand what dynamics to expect from the dynamical system. We have clarified in the figure the steady states available to the bistable switch to make this clearer. In addition, in the new version of the manuscript, we are no longer including the bistable switch in Fig. 3, making more valuable the context of Fig.2. In addition we appreciate the referee's attention to detail; however, upon review, we found that Fig. 2 is indeed cited in the text following the mention of Fig. 1C, adhering to the sequence presented in the manuscript.

> The same problem I honestly encountered with Fig. 3 – it carries a very important message, that global rescaling of protein production and degradation rates of the transcription factors of a given gene regulatory network will preserve the orbit while controlling tempo, but it is unclear whether this is a scheme or these are actual results. This comes from their analytical approach shown in Supplementary – then it should be clearly and more extensively described, and maybe the authors can show to numerical examples (maybe from the models they use later on in the paper).

This comment has also been addressed with the new section 3.1 and its corresponding figure where the concept is applied to the differnetiation of neural progenitors.

> In terms of presentation of the result – I personally find the manuscript confusing to read, whereas I think the results themselves are very strong. For example, the description of Fig. 4 -n I would suggest to invest in relating E to F-G, add the relevant periods and distances on the axes and discuss in greater extent the implication of these findings. Same goes for Fig. 5.

We thank the reviewer for their positive comments regarding the results of the manuscript. We hope that the changes suggested by reviewer 1 have addressed some of the confusing aspects of the manuscript. We have also added extra text in the legend of Fig 4 and in the corresponding text in section 3.2 to relate E to F-G and make clear the connection among the panels.

3 Reviewer 3

> Although the question laid out by the authors is of interest, my evaluation is that the insights delivered by their manuscript remain limited. On the one hand, it is my impres- sion that the definitions of system equivalence and distance measures considered are well defined only for systems with a single, periodic orbit.

It seems there was a misunderstanding regarding our framework's applicability to systems beyond those with a single, periodic orbit. To address this, we have added a new section that specifically illustrates the framework's application to systems without a periodic orbit in which the system evolves towards a single steady state in the scenario of the neural progenitor differentiation.

> How does one define a distance between systems with multiple attractors, like the bistable system in Fig. 3?

Since the distance is only evaluated along the trajectory of gene expression, the number of available steady states does not affect the way that distances are calculated. This is the case of Fig. 2. In addition, this is exemplified in the new neuron differentiation section (3.1) where there can also be multiple attractors. If changes in the parameters change the final attractor of the trajectory, it will automatically result in a larger orbital distance. This is discussed in the new section 3.1.

> How should different trajectories, originating from different initial conditions, be weighted?

In our case we are concerned on how the flow field (dynamical landscape) changes under perturbations of the biochemical parameters. This changes in the landsacpe result in changes in the trajectory given a fixed initial condition. The question raised by the referee, concerning the impact of varying initial conditions rather than biohemical parameters is indeed very interesting. The stability of orbits when initial conditions are perturbed has been extensively explored through the concept of homeorhesis. To reflect on this, we have incorporated adiscussion point on potential

© 2024. Published by The Company of Biologists under the terms of the Creative Commons Attribution License (https://creativecommons.org/licenses/by/4.0/).

future research directions that might compare and contrast the concepts of homeorhesis and orbital equivalence.

> Also, in defining distances, should one allow for a rescaling of gene expression levels, to allow that these could in principle be altered without changing the overall dynamics of a system?

This is a good question from the reviewer. As explained in the paper, we do rescale gene expression levels in our work (paragraph before equation 12), but how and when rescaling should be applied in experimental work will depend on what we understand biologically as ”the same” orbit. We have clarified this idea in that paragraph.

> What about more elaborate mappings in gene expression space that preserve function?

This is a question of what one defines as ‘biological function’, which is an important question. We have added a discussion on the topic, guided by suggestions made by reviewer 1, which justifies our choice of definition and what other possible choices there could be.

> The discussion mentions ”comparing orbits directly from gene expression data”, but no specific discussion of applications to actual experimental data is given.

Specific applications to actual experimental data is a positive consequence of our work, which we aim to explore in future work. It will require an entire paper of its own, which we look forward to working on next.

> I also did not find the examples intended to demonstrate the framework very compelling. The first of what is presented in the abstract as two case studies is just a brief statement of a default model for changes in tempo - the global modulation of all reaction rates (Section 3.1).

We hope that the new section applied to V3 interneuron differentiation (3.1) is more com- pelling and relevant to developmental biology.

> Figure 3 illustrates this with a time-dependent modulation of reaction rates, but would a state-dependent modulation of the rates (mu(x) instead of mu(t)) not be equally relevant, where systems with periodic orbits are concerned?

This is correct and has been added to the sentence to make it clearer.

> The second example is mostly taken from a published study of a particular gene network, and does not appear to me to deliver insights that are particularly new, general, or convincing.

Although the model has been taken from a published study we have used the model in a new context and in a novel way. In fact, one reason the repressilator was chosen was because it is a well-studied regulatory system in synthetic biology. The reduction technique used in Bennet’s paper was introduced in that original study purely to increase the accuracy of the reduced model. We have taken this technique and revealed a deeper meaning of it by examining the global prefactor that it produces, a completely new insight. Since the technique is based on, and is an extension of, quasi-steady state reduction, it is a general result which can be used on any system that allow for time-scale separation. Given that by far the most common way to reduce biological dynamical systems models is by quasi-steady state reduction, we regard this method as general. In addition, the exploration through the new motor neuron example should provide an additional biological example more relevant for the readership of Development.

> To take one example, in their discussion of this system, the authors point to certain combinations of parameters (r_i) as levers to control tempo (Section 3.2); but, if I am not mistaken, the prefactors μ_i from eq. 11, which should vary coherently to preserve orbits, also depend on the system state (on the p_i), thus I am not clear that is choice is so well justified, or how this is supposed to give an analytical bearing on the problem (cf. first paragraph of Section 3.3).

© 2024. Published by The Company of Biologists under the terms of the Creative Commons Attribution License (https://creativecommons.org/licenses/by/4.0/).

We agree with the reviewer that a difficulty with modifying parameters is that it can affect the prefactors in complex ways due to the prefactors depending on the system state. This is the reason why we introduced the concept of prefactor heterogeneity in section 2, and the motivation behind the parameter search in the repressilator example, which would not be necessary if the prefactors scaled homogeneously. The particular choice of r_i comes from the fact that they are the only parameters that appear exclusively in the prefactor. Nevertheless, despite the complicated relation of the prefactors with the system state, we were able to find such a combination, as demonstrated by Fig 5E, which we believe overcomes the potential problem raised by the referee. We understand that this will not be true for other networks, this has been clarified by softening some of the claims in sections 3.2-3.3.

Resubmission

First decision letter

MS ID#: DEVELOP/2024/202950

MS TITLE: A Mathematical Framework for Measuring and Tuning Tempo in Developmental Gene Regulatory Networks

AUTHORS: Charlotte L Manser and Ruben Perez-Carrasco
ARTICLE TYPE: Research Article

I am happy to tell you that your manuscript has been accepted for publication in Development, pending our standard ethics checks.

Reviewer 1

Advance summary and potential significance to field

This manuscript introduces a novel mathematical framework to better understand how the timing of gene expression affects embryo development. Despite similar genetic networks acting in different species, the timing of gene expression can vary greatly. This timing, or "tempo," is critical because it controls the proper development of an organism. While substantial experimental work is being done to decode the molecular mechanisms of tempo, there is a lack of understanding of how existing mathematical models can incorporate tempo without altering the system's dynamics.

The authors bridge this knowledge gap by presenting a mathematical model that associates the functionality of developmental gene programs with their gene expression orbits or landscapes. The key innovation is the ability to view differences in tempo as changes within the dynamical system that do not disrupt its overall patterns or orbits. The proposed framework is designed to predict the molecular mechanisms that control the timing of gene expression through both computational and analytical techniques.

The paper includes two practical examples: a generic gene network model focusing on production and degradation dynamics, applied specifically to neural progenitor cell differentiation, and the repressilator, a classic example of synthetic genetic oscillator.

The significance of this research lies in its potential application: identifying molecular mechanisms that specifically control the timing of gene expression without affecting the developmental trajectory of an organism. Such insights could enable the precise design of biological circuits that maintain desired dynamical behaviors while operating at different tempos, which is a notable step forward in developmental biology and synthetic biology.

Comments for the author

© 2024. Published by The Company of Biologists under the terms of the Creative Commons Attribution License (https://creativecommons.org/licenses/by/4.0/).

The manuscript is relevant, interesting, and clear. Previous Reviewers have made a thorough effort in their reports, and I find the author's responses appropriate and the corresponding modifications of the manuscript, adding depth and clarity. I think this very generic mathematical works play a very interesting role as frameworks for thought for the whole community, and therefore recommend publication in Development.

Reviewer 2

Advance summary and potential significance to field

I thank the authors for the detailed changes to the manuscript in response to the raised comments and suggestions. I find the revised figures much clearer, and the text easier to read. In particular the example of the ventral neural tube model is very helpful to highlight the strengths of the proposed framework. In my opinion, the paper has significantly improved and would be a strong contribution to the conceptual understanding of tempo generation during development.

Comments for the author

/

© 2024. Published by The Company of Biologists under the terms of the Creative Commons Attribution License (https://creativecommons.org/licenses/by/4.0/).
